# A Remarkably Unsymmetric Hexairon Core Embraced by Two High-Symmetry Tripodal Oligo-α-pyridylamido Ligands

**DOI:** 10.1021/acs.inorgchem.3c00808

**Published:** 2023-06-22

**Authors:** Alessio Nicolini, Trey C. Pankratz, Marco Borsari, Rodolphe Clérac, Antonio Ranieri, Mathieu Rouzières, John F. Berry, Andrea Cornia

**Affiliations:** † Department of Chemical and Geological Sciences, University of Modena and Reggio Emilia & INSTM, I-41125 Modena, Italy; ‡ Department of Chemistry, University of Wisconsin−Madison, 1101 University Avenue, Madison, Wisconsin 53706, United States; § Univ. Bordeaux, CNRS, CRPP, UMR 5031, F-33600 Pessac, France; ∥ Department of Life Sciences, University of Modena and Reggio Emilia, I-41125 Modena, Italy

## Abstract

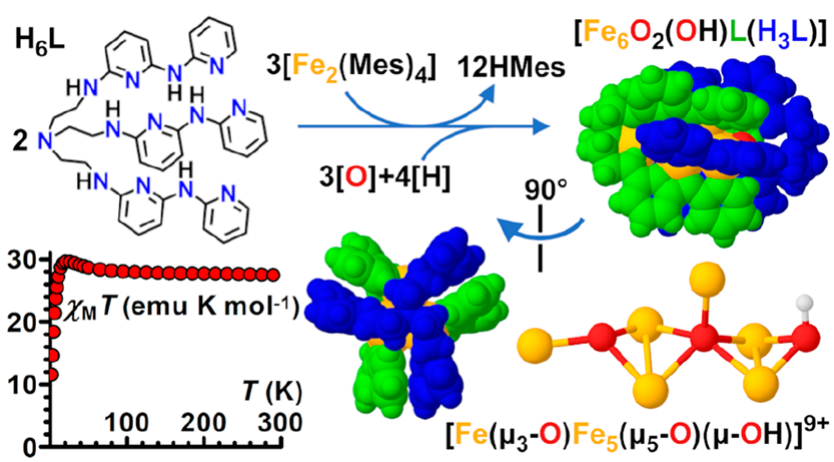

Oligo-α-pyridylamides offer an appealing route to polyiron complexes with short Fe–Fe separations and large room-temperature magnetic moments. A derivative of tris(2-aminoethyl)amine (H_6_tren) containing three oligo-α-pyridylamine branches and 13 nitrogen donors (H_6_L) reacts with [Fe_2_(Mes)_4_] to yield an organic nanocage built up by two tripodal ligands with interdigitated branches (HMes = mesitylene). The nanocage has crystallographic *D*_3_ symmetry but hosts a remarkably unsymmetric hexairon–oxo core, with a central Fe_5_(μ_5_-O) square pyramid, two oxygen donors bridging basal sites, and an additional Fe center residing in one of the two tren-like pockets. Bond valence sum (BVS) analysis, density functional theory (DFT) calculations, and electrochemical data were then used to establish the protonation state of oxygen atoms and the formal oxidation states of the metals. For this purpose, a specialized set of BVS parameters was devised for Fe^2+^–N^3–^ bonds with nitrogen donors of oligo-α-pyridylamides. This allowed us to formulate the compound as [Fe_6_O_2_(OH)(H_3_L)L], with nominally four Fe^II^ ions and two Fe^III^ ions. Mössbauer spectra indicate that the compound contains two unique Fe^II^ sites, identified as a pair of closely spaced hydroxo-bridged metal ions in the central Fe_5_(μ_5_-O) pyramid, and a substantially valence-delocalized Fe^II^_2_Fe^III^_2_ unit. Broken-symmetry DFT calculations predict strong ferromagnetic coupling between the two iron(II) ions, leading to a local *S* = 4 state that persists to room temperature and explaining the large magnetic moment measured at 300 K. The compound behaves as a single-molecule magnet, with magnetization dynamics detectable in zero static field and dominated by an Orbach-like mechanism with activation parameters *U*_eff_/*k*_B_ = 49(2) K and τ_0_ = 4(2) × 10^–10^ s.

## Abstract 2

With their interdigitated branches and 26 nitrogen donors, two tripodal oligo-α-pyridylamido ligands define a trigonally symmetric coordination nanospace that accommodates a mixed valence iron cluster of much lower symmetry. Short metal−metal distances coexist with oxygen bridges in this compound, which exhibits a large room-temperature magnetic moment and behaves as a single-molecule magnet.

## Introduction

**Chart 1:**
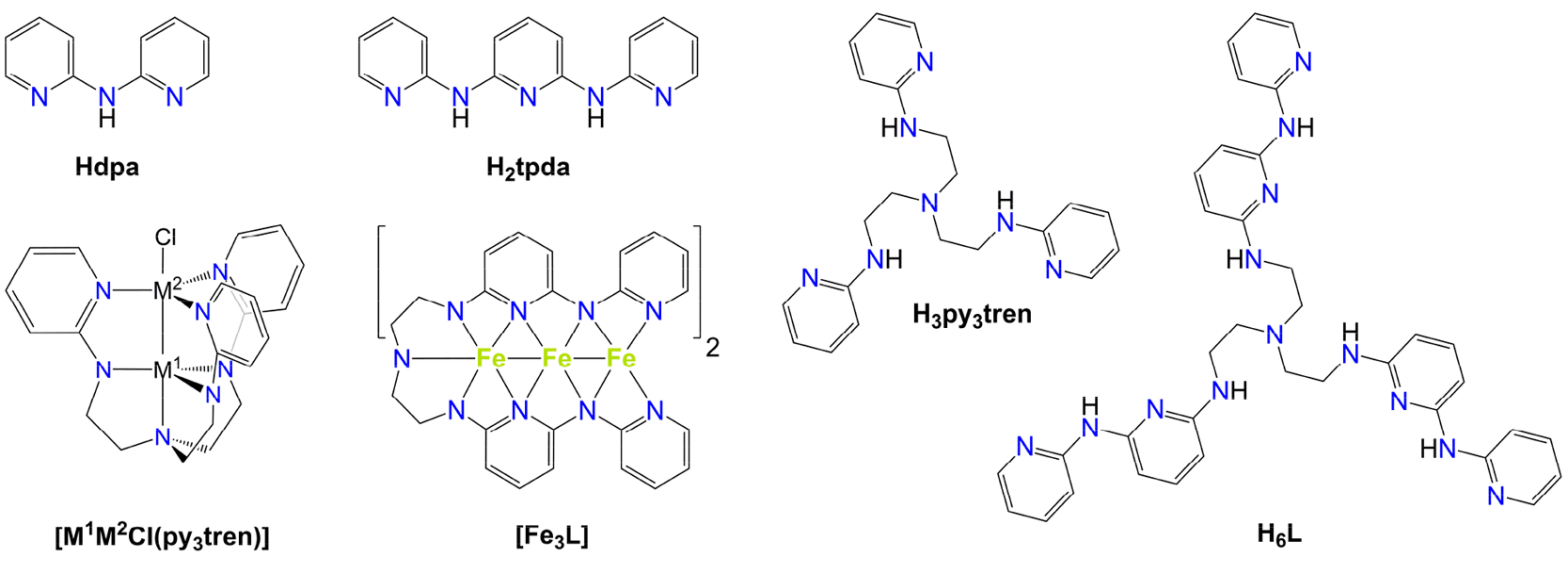
Structures of Hdpa, H_2_tpda, H_3_py_3_tren, H_6_L, and Complexes [M^1^M^2^Cl(py_3_tren)] (M^*i*^ = Fe^2+^, Co^2+^, or Mn^2+^)^27^ and [Fe_3_L]

A frontier in the chemistry of metal–metal bonds is the deliberate construction of compounds containing extended metal atom chains (EMACs).^1−9^ Oligo-α-pyridylamido ligands have been foundational to this area of research. The simplest example is the anion of di(pyridin-2-yl)amine [Hdpa (Chart 1)],^10^ which is known to support more than 300 EMAC entries in the Cambridge Structural Database (CSD).^11^ Also shown in Chart 1 is H_2_tpda, an extended proligand whose dianionic form can afford pentametallic EMACs.^12^

Although extremely rare, iron-based EMACs are of special significance, because short Fe–Fe separations can stabilize high-spin (HS) states that persist to room temperature.^13−15^ In 2018, we discovered that tpda^2–^ can support linear tetrairon(II) chains of the type [Fe_4_(tpda)_3_X_2_] with X = Cl (**1**) or Br (**2**).^16,17^ In these EMACs, the three tpda^2–^ ligands wrap around the metal atom chain to give a helical structure with 3-fold symmetry.^a^ This type of symmetry is comparatively rare in EMAC structures and was previously encountered only in copper(I) chemistry.^18,19^ It is also rare among metal–metal bonded dimers supported by N donor ligands, with [Fe_2_(form)_3_] and [Co_2_(form)_3_] representing early examples (Hform = *N*,*N′*-diphenylformamidine),^20−22^ which have been followed by a spectacular Cr_2_ compound having a Cr–Cr quintuple bond.^23−26^ However, the incorporation of only four of five possible metals in **1** and **2** results in large Fe–Fe separations (2.94–2.99 Å) and weak magnetic interactions. More recently, Guillet and co-workers used a sterically hindered derivative of 2,6-diaminopyridine to enforce much shorter Fe–Fe distances (2.44 Å) in a trigonally symmetric triiron(II) EMAC, which in fact exhibits a well-isolated *S* = 6 state.^13^

In 2014, Bill, Gagliardi, Lu, and co-workers utilized H_3_py_3_tren, a triply arylated derivative of tris(2-aminoethyl)amine (H_6_tren), to promote short M–M distances (2.29–2.53 Å) in complexes [M^1^M^2^Cl(py_3_tren)] with M^*i*^ = Fe^2+^, Co^2+^, or Mn^2+^ (Chart 1).^27^ Building off of this precedent, we imagined that an extended version of the H_3_py_3_tren proligand could be used to support trigonally symmetric EMACs. Therefore, we designed, synthesized, and isolated in good yield (∼60%) the new polynucleating tripod H_6_L (Chart 1), which contains 13 nitrogen donors of four different types, and one more α-pyridylamino unit per branch than H_3_py_3_tren.^28^

In this work, we report our first attempt to use H_6_L to form iron(II)-based EMACs. To our surprise, we failed to isolate the expected neutral complex [Fe_3_L] (Chart 1) or longer species. Instead, we reproducibly obtained a remarkably unsymmetric hexairon–oxo core hosted in a 3-fold-symmetric organic nanocage provided by two tripodal ligands with interdigitated branches. Combining elemental analysis (EA), electrospray ionization mass spectrometry (ESI-MS), X-ray crystallography, electrochemistry, and Mössbauer spectroscopy with density functional theory (DFT) and bond valence sum (BVS) calculations, we reached the formula [Fe_6_O_2_(OH)(H_3_L)L] (**3**) for this compound. On the basis of our current understanding, in **3** a central square-pyramidal Fe_5_(μ_5_-O) core is embraced by two organic ligands (H_3_L^3–^ and L^6–^) and coordinated by two additional oxo groups, one of which is protonated. Similar square-pyramidal Fe_5_(μ_5_-O) motifs have been observed in previous studies.^29−31^ The fully deprotonated L^6–^ ligand hosts a sixth Fe center in its tren-like pocket, while the same coordination site in H_3_L^3–^ remains unmetalated. The compound nominally contains four Fe^II^ ions and two Fe^III^ ions with substantial valence delocalization. It has a large room-temperature magnetic moment (14.8 μ_B_) and exhibits slow relaxation of its magnetization at low temperature, detectable even in zero static field.

## Experimental Section

### General Procedures

All synthetic operations involving iron complexes were carried out inside an MBraun UNIlab glovebox under an inert and controlled dinitrogen atmosphere, continuously purified over activated charcoal, molecular sieves, and a copper oxygen scavenger (<1 ppm H_2_O and O_2_). All chemicals were of reagent grade and used as received, unless otherwise noted. Anhydrous toluene and 1,4-dioxane were purchased and used exclusively for operation inside the glovebox. Thf and Et_2_O were predried over KOH^32^ and CaCl_2_,^33^ respectively, and subsequently distilled from their sodium diphenylketyl solutions before use. All of the solvents (including thf-*d*_8_) used inside the glovebox were deoxygenated through three freeze–pump–thaw cycles and stored over 4 Å molecular sieves (except for EtOH, which was simply stored over 3 Å molecular sieves). H_6_L was prepared by heating together *N*-(6-fluoropyridin-2-yl)pyridin-2-amine, H_6_tren, and Cs_2_CO_3_ at 130 °C for 3 days under solvent-free conditions, as described previously.^28^ The purified material was isolated as H_6_L·0.44EtOH. [Fe_2_(Mes)_4_] was synthesized from Fe_4_Cl_8_(thf)_6_^34^ and MesMgBr in thf/1,4-dioxane.^35^ Tetra-*n*-butylammonium tetrafluoroborate (TBATFB) used as the base electrolyte for electrochemical studies was recrystallized twice from EtOH/Et_2_O [1:1 (v/v)] at −50 °C. The crystalline solid was filtered, washed with Et_2_O, and dried under vacuum.^33^

Unless otherwise noted, all characterization data were collected with strict exclusion of air under a dinitrogen atmosphere. The CHN content was determined using a ThermoFisher Scientific Flash 2000 analyzer and implied the unavoidable exposure of the sample to the air for 30–60 s, whereupon it immediately turned from dark red to black. The Fe content was determined by complexometric titration on a sample of **3** accurately weighed inside the glovebox and digested with HNO_3_ and H_2_O_2_ under aerobic conditions [titrant, Na_2_EDTA·2H_2_O, standardized using Pb(NO_3_)_2_ as a primary standard; indicator, xylenol orange].^36^ ESI-MS measurements were conducted on a model 6310A Ion Trap LC-MS(n) instrument (Agilent Technologies) by direct infusion of thf solutions, in positive ion mode. The electronic spectra for the thf solution were recorded up to 2000 nm on a double-beam ultraviolet–visible–near infrared (UV–vis–NIR) Jasco V-570 spectrometer, using a quartz cuvette sealed with an airtight Teflon cap (optical path length *l* of 0.1 cm). The ^1^H nuclear magnetic resonance (NMR) spectrum was recorded at 298 K in thf-*d*_8_ using a Bruker Avance 400 FT-NMR spectrometer (400.13 MHz) and an airtight Young-valved NMR tube. The chemical shifts are expressed in parts per million downfield from Me_4_Si as the external standard, by setting the residual CDH(3,4) signal of thf-*d*_8_ at 1.72 ppm.^37^ Spectra were analyzed with TopSpin version 4.0.6.^38^ The following abbreviations were used in reporting spectroscopic data: br, broad; sh, shoulder.

### Synthesis of [Fe_6_O_2_(OH)(H_3_L)L] (**3**)

Inside a glovebox, [Fe_2_(Mes)_4_] (265.6 mg, 0.4514 mmol) and H_6_L·0.44EtOH (184.8 mg, 0.2742 mmol) were refluxed together in toluene (7 mL) for 3 h. During the reflux, the suspension progressively turned from orange to dark red/brown. The reaction mixture was allowed to cool to room temperature, and the solution was then eliminated by filtration through a fritted glass filter (G4 porosity). The dark red solid was washed with fresh toluene (1 mL) and extracted with thf (4 × 10 mL). One additional overnight extraction was performed with 20 mL of thf. The combined red extracts were evaporated under vacuum, to give the product as a red solid (134.0 mg, 0.07964 mmol, 58.1%). Anal. Calcd for **3**: C, 51.40; H, 4.19; N, 21.64; Fe, 19.91. Calcd for **3**·0.4C_7_H_8_: C, 52.25; H, 4.29; N, 21.18; Fe, 19.49. Found: C, 52.44; H, 4.18; N, 21.42; Fe, 19.7. This red solid can be recrystallized from thf/Et_2_O by slow liquid diffusion to afford small, dark red, trapezoidal plates of **3** (recrystallization yield of ∼50%). For all of the following characterizations, except X-ray crystallography, crystals of **3** were removed from the mother solution, washed with a few drops of Et_2_O, and dried well under vacuum. Anal. Calcd for **3**·0.3Et_2_O: C, 51.57; H, 4.32; N, 21.36. Found: C, 51.86; H, 4.12; N, 21.43. ^1^H NMR (thf-*d*_8_, 298 K, 400.13 MHz): δ 3.38 (4H, br, Et_2_O, CH_2_), 1.11 (6H, br, Et_2_O, CH_3_). ESI-MS (thf, positive ion mode): *m*/*z* 1680.2–1681.2 ([Fe_6_O_3_H_2_L_2_]^+^ and [Fe_6_O_3_H_3_L_2_]^+^, 0.8:0.2 ratio, 100), 1626.3–1627.3 ([Fe_5_O_3_H_4_L_2_]^+^ and [Fe_5_O_3_H_5_L_2_]^+^, 0.9:0.1 ratio, 10), 850.1 ([Fe_3_ClL]^+^, 3). UV–vis–NIR (thf, 1.43 × 10^–4^ M): λ_max_ (nm) [ε (M^–1^ cm^–1^)] 242 (1.03 × 10^5^), 268 (sh), 341 (6.83 × 10^4^), ∼380 (sh), ∼500 (sh).

### X-ray Crystallography

A platelike crystal of **3** was covered with NVH immersion oil (Jena Bioscience), which efficiently protects the compound against degradation. Subsequently, it was mounted on a MiTeGen Microloop and transferred to a Bruker-Nonius X8APEX diffractometer equipped with a Mo Kα generator, an area detector, and a Kryoflex liquid dinitrogen cryostat for the collection of data at 115(2) K. Matrix frames and data were acquired using APEX2 version 1.0-22.^39^ Data reduction was achieved with SAINT version 7.06A^39^ and was followed by multiscan absorption correction applied with SADABS version 2.10.^39^ SUPERFLIP^40,41^ and SHELXL-2014/7,^42^ implemented in the WINGX version 2020.1 suite,^43^ were used for structure solution and refinement on *F*_o_^2^, respectively. Due to the massively disordered structure, the measured crystal, and likewise all tested samples, was weakly diffracting and data collection was extended to a resolution of 0.84 Å (2θ_max_ = 50.0°) to give an ⟨*I*/σ(*I*)⟩ of 6.74. The unit cell metrics and the symmetry of the diffraction pattern were consistent with the trigonal crystal system, with *R*(merge) values of 0.078, 0.073, and 0.048 in Laue classes 31*m* (hex), 3 (hex), and 1, respectively. Systematic absences were found to affect *hhl* reflections, which had ⟨*I*/σ(*I*)⟩ values of −0.16 for odd *l* values (320 reflections) and 10.29 for even *l* values (311 reflections), strongly suggesting extinction symbol *P*--*c* and space group *P*31*c* (acentric) or *P*31*c* (centric). Structure solution in the centric space group followed by a Fourier synthesis gave the positions of all non-hydrogen atoms, which were treated anisotropically except for Fe3. Hydrogen atoms were added in calculated positions with *U*(H) = 1.2*U*_eq_(C,N), including the half-occupancy secondary NH hydrogen atoms of the 50% unmetalated tren-like pocket, which were directly located in Δ*F* maps. Although *E* statistics favor a noncentrosymmetric structure, refinement in space group *P*31*c* is clearly overparametrized. Disorder persists even in this acentric space group, showing that it is not caused by a misassigned space group.

The structure of the central core was best revealed by a Fourier synthesis with phases based on the organic ligands alone (Figure S4). The most intense peak (Q1 = 8.11 *e* Å^–3^) and its symmetry equivalents define a slightly distorted trigonal prism, with shortest separations of 2.67 and 3.12 Å. Each longer edge of the prism is capped by an additional peak (Q2 = 5.56 *e* Å^–3^) lying exactly on a 2-fold axis 1.61 Å from the vertices. Other significant electron density residuals were found on the 3-fold axis, namely in the tren-like pockets of the ligands (Q3 = 4.57 *e* Å^–3^), at the center of the prism (Q4 = 4.52 *e* Å^–3^), and in triangular-face capping positions (Q5 = 3.93 *e* Å^–3^). Q1 and Q2 are at coordination-bond distances from N atoms (2.02–2.33 Å) as well as from Q4 and Q5 (1.92–2.18 Å), suggesting that the former are partially occupied metal sites, while the latter are O atoms. The Q1–Q2 distance is indeed unphysically short for a metal–metal contact and requires the central core to be severely disordered. Full anisotropic refinement with the assignments suggested above (Q1 = Fe1, Q2 = Fe2, Q3 = Fe3, Q4 = O1, and Q5 = O2) and free site occupancy factors (SOFs) gave SOF/*U*_iso_(Å^2^) = 0.634(6)/0.0424(6), 0.310(6)/0.0303(15), and 0.440(10)/0.096(3) for Fe1, Fe2, and Fe3, respectively; SOF/*U*_iso_(Å^2^) = 0.92(4)/0.030(4) and 1.09(3)/0.047(3) for O1 and O2, respectively. These results suggest 2:1 occupancies for Fe1 and Fe2, approximately half-occupancy for Fe3, and full occupancies for O1 and O2. Anisotropic displacement parameters (ADPs) had physically reasonable values except for Fe3, which displayed a very oblate ellipsoid. The ADPs for the secondary N donors of the tren-like pocket were also distinctly elongated toward the metal, suggesting an off-axis position for Fe3. Slant plane Δ*F* maps normal to the trigonal axis indeed confirmed a pronounced 3-fold modulation of electron density around Fe3, but not around O1 and O2. Fe3 was then allowed to move off the 3-fold axis and was treated isotropically, affording SOF/*U*_iso_(Å^2^) = 0.141(3)/0.034(2) for Fe3 and virtually unvaried parameters for the remaining core atoms. This result indicates clearly that, independent of how Fe3 is modeled, the tren-like pocket of the ligand is only approximately 40–50% occupied. Because both EA and ESI-MS consistently indicate six metal centers per molecule, fixed occupancies of ^2^/_3_, ^1^/_3_, and ^1^/_6_ were assigned to Fe1, Fe2, and Fe3, respectively, thereby imposing half-occupancy of the tren-like pocket. Unit occupancies were instead used for O1 and O2. Notice that restrained anisotropic refinement (ISOR card) was also attempted for Fe3, yielding a very elongated displacement ellipsoid but minimal changes in geometry and bond precision. For this reason, isotropic refinement was preferred. The 2:1 occupancies of Fe1 and Fe2 suggest a distorted square-pyramidal Fe_5_(μ_5_-O) core orientationally disordered around the trigonal axis, with Fe2 as the apical site. BVS calculations (Table S2) support the assignment of central oxygen O1 as a μ_5_-O^2–^ ligand. Furthermore, they are consistent with O2 being a μ_3_-O^2–^ group when the neighboring tren-like pocket is metalated, and a μ-OH^–^ group otherwise. The severely disordered core requires the electron density of the hydroxide hydrogen atom to be spread over six positions, thus explaining why it could not be located in Δ*F* maps. For this reason, the OH^–^ hydrogen atom was not included in the refinement. The rather high final *R* indices at least partially reflect unaccounted for electron density located in the solvent-accessible voids of the structure. In fact, ^1^H NMR spectroscopy (Figure S3) indicates that crystals of **3** retain traces of Et_2_O even after prolonged treatment in vacuum, in accordance with EA. The solvent-accessible voids in the structure were calculated using the SQUEEZE command^44^ implemented in PLATON version 90622.^45^ This calculation showed that four solvent-accessible voids exist per unit cell, namely two 216 Å^3^ voids (each containing 40 electrons) and two 126 Å^3^ voids (each containing 43 electrons). The latter host the highest-electron density residuals (2.0 *e* Å^–3^) in the final Δ*F* map. Therefore, each of these voids can in principle host one Et_2_O molecule, which contains 42 electrons and has a van der Waals volume of ∼86.6 Å^3^ (calculated using the method proposed by Abraham et al.).^46^ We found that the use of solvent-corrected *hkl* data lowers the *R*_1_ factor for reflections with *I* ≥ 2σ(*I*) from 8.5% to 5.6%. However, differences in molecular geometry are insignificant, and we decided to base the final refinement on pristine, uncorrected data. Graphics utilized ORTEP-3 for Windows version 2014.1,^43^ POV-Ray for Windows version 3.7,^47^ and Olex2-1.5.^48^

### Mössbauer Spectroscopy

Solid state Mössbauer spectra of **3** were recorded at 77 and 10 K with a SEE Co model W304 resonant γ-ray 1024 channel spectrometer with a ^57^Co on Rh foil source. The velocity range used was ±4 mm s^–1^, and the experimental spectra were referenced to α-Fe foil at room temperature. Data were collected with the sample under vacuum. Mössbauer data were fitted using an adaptive nonlinear least-squares algorithm developed by Dennis et al. and available within the WMOSS4F software.^49^ Three quadrupole doublets were used to fit the experimental data. The following parameters were fitted for each quadrupole doublet: the isomer shift (δ), which is highly diagnostic of oxidation state; the quadrupole splitting (Δ*E*_Q_), which reports on the electric field gradient at the Fe site; and the line width (i.e., the full width at half-maximum, Γ_fwhm_). The relative areas of the subspectra were either allowed to vary or fixed to 4:1:1, as initially suggested by crystallographic symmetry with four basal sites and two additional independent sites (apical and pocket sites). However, we note that the DFT-optimized model deviates from this simplistic assignment and suggests that it is two of the basal sites that have the unique Mössbauer parameters.

### Magnetic Measurements

The magnetic measurements were obtained with a Quantum Design MPMS-XL SQUID magnetometer and a PPMS-9 susceptometer. The MPMS-XL instrument works between 1.85 and 400 K with applied static fields (*H*) ranging from −70 to 70 kOe. The sample for magnetic measurements was prepared with strict exclusion of dioxygen and moisture in a glovebox under argon. A polycrystalline sample of **3** (7.1 mg) was covered with Paratone oil (10.2 mg) to avoid magnetic torqueing and introduced into a sealed polypropylene bag (3 cm × 0.5 cm × 0.02 cm; 20.9 mg). Prior to the experiments, the field-dependent magnetization was measured at 100 K to exclude the presence of bulk ferromagnetic impurities. All magnetic data were corrected for the sample holder contribution and reduced using the molar mass and intrinsic diamagnetism appropriate for **3**. Any minority species possibly co-crystallized with **3** was neglected. The direct current (dc) magnetic susceptibility (χ) was obtained as *M*/*H* from magnetization (*M*) measurements at 1 and 10 kOe in the temperature range of 1.85–300 K. Isothermal magnetization data were also recorded between 1.86 and 8 K in fields up to 70 kOe. Above 1.86 K, no hysteresis effects were observed in the field dependence of the magnetization for field sweep rates between ∼50 and ∼600 Oe min^–1^. The alternating current (ac) susceptibility measurements down to 1.9 K were performed using an oscillating field of 1–6 Oe for frequencies (ν) from 10 Hz to 10 kHz and applied static fields from 0 to 20 kOe (PPMS-9). In the available temperature and frequency ranges, the sample displayed slow relaxation of the magnetization that could be observed in zero and in finite applied dc fields. All ac measurements were fitted to the generalized Debye model (using χ′ and χ″ vs ν data) to extract the characteristic relaxation time (τ), the α parameter describing the width of the distribution of relaxation times, and the values of χ_∞_′ and χ_0_′−χ_∞_′.

### Electrochemistry

Cyclic voltammetry (CV) curves were recorded in thf using a PARSTAT 2273 potentiostat/galvanostat (Princeton Applied Research, Oak Ridge, TN). Experiments at different scan rates (*v* = 0.02–1 V s^–1^) were carried out using a cell for small-volume samples (∼3 mL). A Pt ring, a Ag wire, and a 1 mm diameter glassy carbon (GC) disk (Princeton Applied Research) were used as the counter, quasi-reference, and working electrodes, respectively. The GC electrode was cleaned as previously reported.^50^ For all experiments, the potential of the quasi-reference electrode was calibrated against the ferrocenium/ferrocene (Fc^+^/Fc) redox couple.^51^ All of the reported potential values are also referenced to the Fc^+^/Fc redox couple. To avoid degradation of the complex, all of the measurements were performed in an MBraun UNIlab glovebox under dinitrogen at −10 °C. The concentrations of **3** and the base electrolyte (TBATFB) were 0.25 mM and 0.05 M, respectively. To minimize the ohmic drop between the working and reference electrodes, a careful feedback correction was applied. All of the formal potential values (*E*°′) were calculated as the semisum of the cathodic and anodic peak potentials, i.e., *E*°′ = (*E*_pc_ + *E*_pa_)/2. The dependence of Δ*E*_p_ = *E*_pa_ – *E*_pc_ on *v* allowed us to obtain the standard heterogeneous electron transfer (ET) rate constant *k*_ET_,^52^ which is the ET rate constant measured at the formal potential *E*°′. The experiments were repeated at least four times, and the *k*_ET_ values obtained were found to be reproducible within 6%. When necessary, signal deconvolution was performed using the EG&G Condecon Software Package.

### Conductivity Measurements

Conductivity measurements were carried out in thf with a CRISON (model *micro*CM2201) conductivity meter using a cell of constant 1.14 cm^–1^ with a precision of 0.5%. They were made in a thermostatic bath at different temperatures (from −23 to 2 °C) kept constant within ±0.1 °C; the concentration of **3** was 0.25 mM. The nature of the complex (neutral or ionic) was determined by comparing its molar conductivity values with those of ionic complexes measured in the same solvent and under the same conditions.

### DFT Calculations

The initial atomic coordinates used in DFT studies were obtained from X-ray diffraction data, omitting crystallographic disorder and adding a hydrogen atom to either O2 or O2^III^ to give model **DFT1** or **DFT2**, respectively. All hydrogen atom positions were then optimized in **DFT1** and **DFT2** while the positions of all heavier atoms were held fixed. In model **DFT3**, all atomic positions were fully optimized. All calculations were performed using the ORCA 4.2.1 software package using unrestricted Kohn–Sham (UKS) DFT.^53^ The def2-SVP basis set was used on all atoms except Fe, for which the def2-TZVP basis set was used.^54^ The numerical grid was increased to “Grid 4” in ORCA notation. Calculations on **DFT1** and **DFT2** were performed using the B3LYP functional with dispersion correction D3.^55,56^ Calculations on **DFT3**, including the evaluation of Mössbauer parameters, were performed using the BP86 functional.^55,56^ In addition, the NBO package was employed in **DFT3** to obtain the natural population values.^57^ The broken-symmetry single-point calculations on **DFT4** were also performed using BP86. Additionally, the resolution of identity (RI) approximation was applied to all of the calculations.^58^

## Results and Discussion

### Synthesis and Solution Studies

H_6_L (Chart 1) was designed with the idea of preparing stable arrays of three or more metal ions, wrapped by its fully deprotonated Hdpa-like branches and arranged linearly to form new EMACs. In an attempt to access neutral triiron(II) species [Fe_3_L] (Chart 1), we followed a synthetic procedure that is similar to that leading to halide-terminated iron(II) EMACs **1** and **2** (eq 1),^16,17^ but omitting the metal halide FeX_2_ precursor (eq 2).
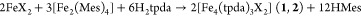
1
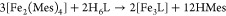
2

Therefore, H_6_L was admixed with a slight excess of [Fe_2_(Mes)_4_] (1:1.6 molar ratio) in toluene, and the mixture was heated to reflux for 3 h in a glovebox (HMes = mesitylene). This reaction, in which the organoiron compound serves both as a metal source and as a strong base, yielded a dark red solid that was separated by filtration and extensively extracted with thf. Evaporation of the solvent led to a red, powdery solid that was characterized by EA, ESI-MS, and UV–vis–NIR spectroscopy (see below). Obtaining X-ray quality crystals of this material proved to be extremely challenging. Many recrystallization attempts were conducted on the basis of slow liquid or gas diffusion of Et_2_O, *n*-hexane, or toluene into thf, 1,4-dioxane, or CH_3_CN solutions, which mostly gave only spherulites of a very dark microcrystalline material. However, slow liquid diffusion of Et_2_O into a thf solution afforded dark red plates, which are exceedingly sensitive to air but sufficiently stable to be handled in immersion oil. The compound was structurally validated by X-ray crystallography (see below), and the main species was identified as the hexairon complex [Fe_6_O_2_(OH)(H_3_L)L] (**3**), containing unexpected oxygen atoms from adventitious sources. In a similar attempt to prepare triiron(II) EMACs supported by dpa^–^ ligands, Cotton et al. reacted FeCl_2_ with Lidpa but failed to obtain the desired linear compound. Instead, they isolated complex [Fe_4_(μ_4_-O)(dpa)_6_] (**4**) with a tetrahedral [Fe_4_(μ_4_-O)]^6+^ unit [the corresponding tetramanganese(II) complex was also obtained starting from MnCl_2_]. The authors attributed the central oxygen atom to a partial hydrolysis of the LiMe reactant used to prepare Lidpa.^59^ McKenzie and co-workers reported the occasional low-yield isolation of **4** co-crystallized with the diiron(II) species [Fe_2_(dpa)_3_Cl] upon reacting [Fe_2_(Mes)_4_]·Et_2_O with Hdpa in toluene, the O^2–^ and the Cl^–^ ligands both being in this case adventitious.^60^ In contrast with these previous reports, compound **3** was reproducibly obtained in two different laboratories (Modena and Madison) and with significant yields, as described by eq 3.
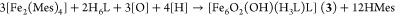
3

The ESI-MS and UV–vis–NIR spectra in thf suggest that the powder and the crystals are the same compound (Figures S1 and S2), though samples in solution are NMR silent (Figure S3). The ESI-MS peaks with *m*/*z* >1500 in the positive ion spectra (Figure S1) can be attributed to species containing two tripodal ligands, three oxygen atoms, a penta- to heptairon core, and a variable degree of protonation ([Fe_*n*_O_3_H_*p*_L_2_]^+^ with *n* = 5, 6, or 7 and *p* = 1–5). Both spectra are dominated by a strong peak around *m*/*z* 1680, whose isotopic pattern can be well simulated as a linear combination of contributions from [Fe_6_O_3_H_2_L_2_]^+^, [Fe_6_O_3_H_3_L_2_]^+^, [Fe_6_O_3_H_4_L_2_]^+^, and [Fe_6_O_3_H_5_L_2_]^+^ (though with different combination coefficients for the powder and the crystals). Minority signals at *m*/*z* 1626 and 1735 are well simulated by penta- and heptanuclear species, respectively ([Fe_5_O_3_H_4_L_2_]^+^ + [Fe_5_O_3_H_5_L_2_]^+^; [Fe_7_O_3_HL_2_]^+^). Below *m*/*z* 1500, the strongest peaks are from [Fe_3_ClL]^+^ (*m*/*z* 850) and [Fe_3_L]^+^ (*m*/*z* 815), with the former presumably reflecting chloride traces from the synthesis of [Fe_2_(Mes)_4_] or adventitious chloride ions in the mass spectrometer.

The UV–vis–NIR spectrum of crystalline **3** dissolved in thf (Figure S2) presents two intense absorptions with maxima (λ_max_) at 242 and 341 nm, along with three shoulders around 268, ∼380, and ∼500 nm. The powder sample exhibits remarkably similar spectral features. As long as contact with air is rigorously excluded, these spectra do not change over long time periods, suggesting that the complex is highly stable in thf solution.

### X-ray Crystallography

**Figure 1:**
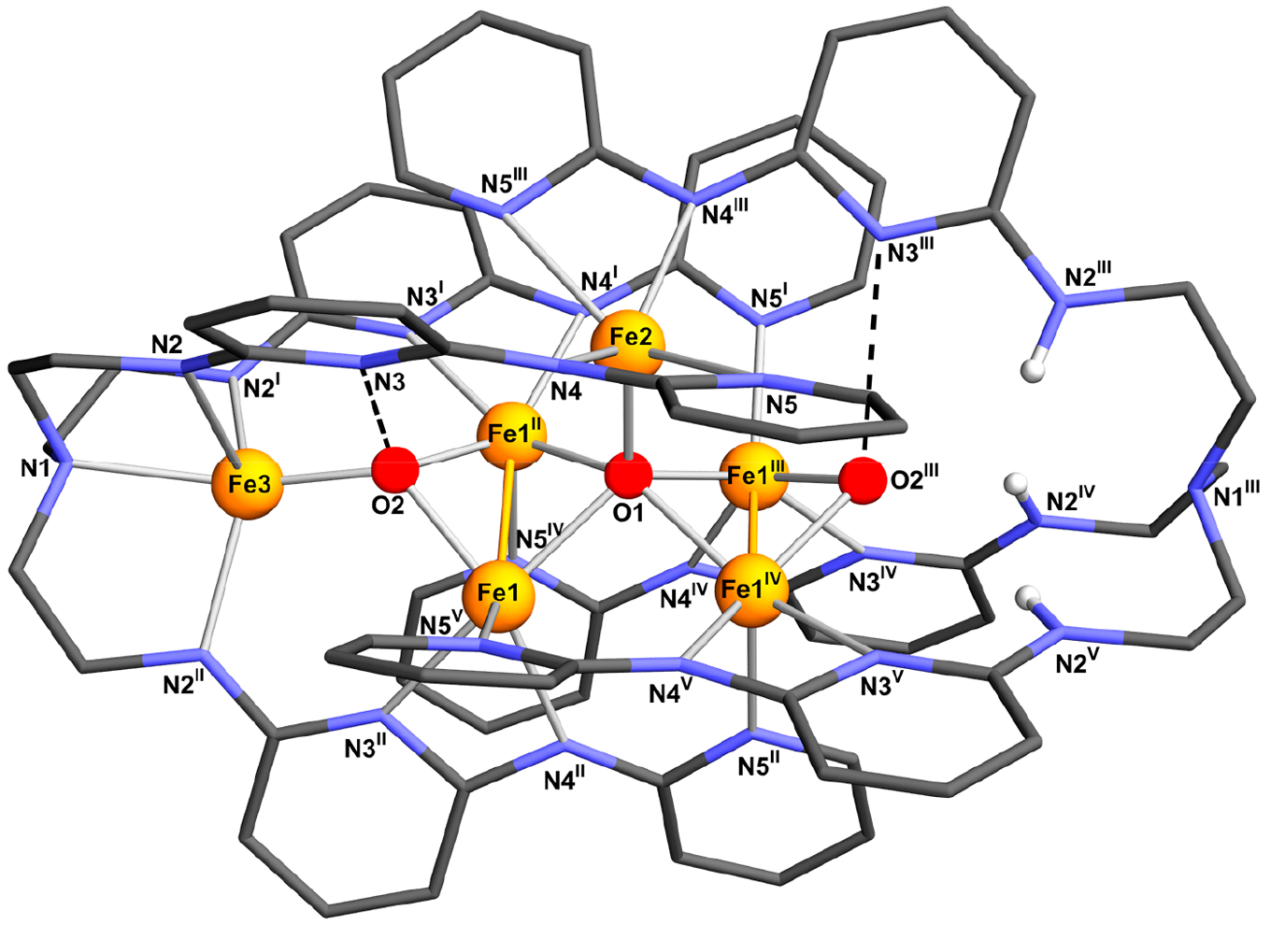
Molecular structure of **3**. The organic ligands are represented with the wireframe model, while Fe and O atoms are drawn using a ball-and-stick model (an ellipsoid representation can be found in Figure S5). The dashed lines connecting O2 and O2^III^ with a N_py_ donor represent possible OH···N_py_ hydrogen bonds. Color code: orange, Fe; red, O; blue, N; dark gray, C; light gray, H. Only the hydrogen atoms bonded to N2^III^, N2^IV^, and N2^V^ are displayed in the figure. Symmetry codes: I = 1 – *x* + *y*, 1 – *x*, *z*; II = 1 – *y*, *x* – *y*, *z*; III = 1 – *x* + *y*, *y*, 0.5 – *z*; IV = *x*, *x* – *y*, 0.5 – *z*; V = 1 – *y*, 1 – *x*, 0.5 – *z*.

Although crystals of **3** are very weak diffractors, a single-crystal X-ray investigation was successfully completed at 115(2) K (Table S1). The final structural model is depicted in Figure 1 and Figure S5 while selected interatomic distances and angles are listed in Table 1. The complex is located on a crystallographic site with *D*_3_ symmetry in space group *P*31*c* (*Z* = 2) and contains two tripodal ligands with imposed 3-fold symmetry and interdigitated branches (Figure 1). All branches are helically wrapped around the 3-fold axis, with ∼25° angles between the mean planes of adjacent pyridine rings. Because the two ligands are symmetry-related by the three dyads orthogonal to the 3-fold axis, they have the same handedness. However, the space group is centrosymmetric and the crystal thus contains both enantiomers in a 1:1 proportion. The distance of the tertiary N atoms (N1 and N1^III^) from the center of the structure is 6.738(9) Å, and the molecular size exceeds 1.6 nm.

**Table 1:** Selected Interatomic Distances and Angles in **3**^a^

distances (Å)		angles (deg)	
Fe1–Fe1^II^	2.629(2)	Fe1–Fe1^II^–Fe1^III^	84.85(4)
Fe1–Fe1^IV^	3.028(3)	Fe1^II^–Fe1–Fe1^IV^	93.065(13)
Fe1–Fe2	3.451(3)	Fe1–O1–Fe1^II^	76.10(5)
Fe1^II^–Fe2	3.232(3)	Fe1–O1–Fe1^IV^	90.47(7)
Fe1–O1	2.1327(13)	Fe1–O1–Fe2	116.18(4)
Fe1–O2	1.997(4)	Fe1^II^–O1–Fe2	105.25(4)
Fe1–N3^II^	2.151(5)	Fe1–O2–Fe1^II^	82.3(2)
Fe1–N4^II^	2.129(5)	N3^II^–Fe1–N4^II^	63.2(2)
Fe1–N5^V^	2.149(5)	N3^II^–Fe1–N5^V^	97.1(2)
Fe2–O1	1.931(3)	N4^II^–Fe1–N5^V^	101.5(2)
Fe2–N4	2.019(5)	N4–Fe2–N5	62.3(2)
Fe2–N5	2.331(5)	N4–Fe2–N5^III^	98.9(2)
Fe3–O2	1.851(8)	N1–Fe3–O2	158.4(3)
Fe3–N1	2.162(10)	N1–Fe3–N2	70.45(18)
Fe3–N2	2.679(8)	N1–Fe3–N2^I^	81.1(3)
Fe3–N2^I^	2.180(12)	N1–Fe3–N2^II^	82.3(3)
Fe3–N2^II^	2.129(11)		
O1···O2	2.796(7)		
O1···N1	6.738(9)		
O2···N3	2.979(5)		

a Symmetry codes: I = 1 −*x* + *y*, 1 – *x*, *z*; II = 1 −*y*, *x* – *y*, *z*; III = 1 −*x* + *y*, *y*, 0.5 – *z*; IV = *x*, *x* – *y*, 0.5 – *z*; V = 1 −*y*, 1 – *x*, 0.5 – *z*.

**Figure 2:**
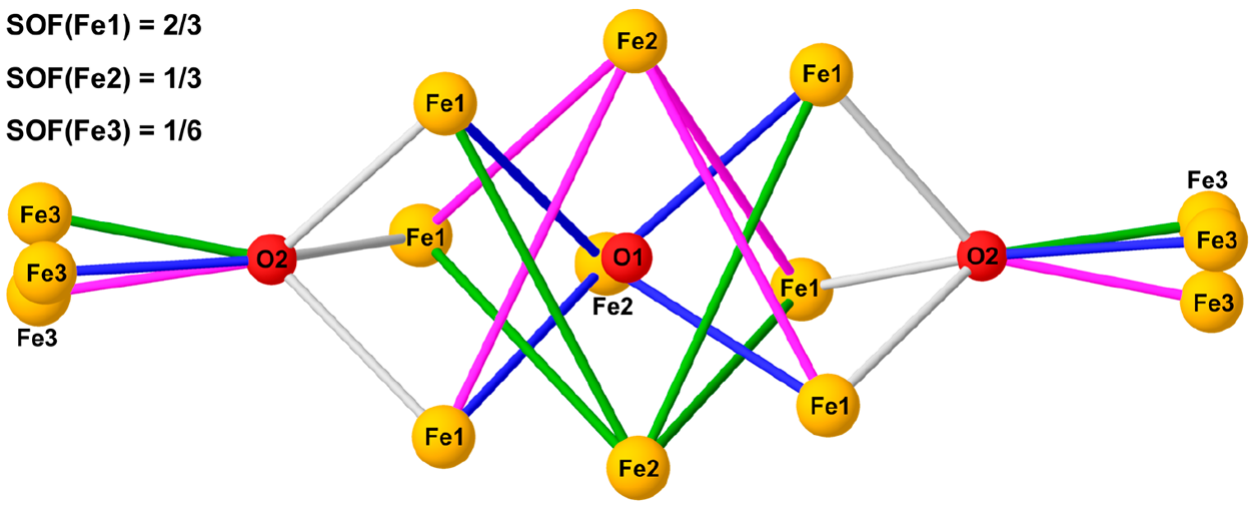
Orientational disorder of the metal core of **3** around the crystallographic 3-fold axis (ball-and-stick model; SOF, site occupancy factor). O1 and O2 have full occupancy (SOF = 1) and lie on the *D*_3_ symmetry site and on the 3-fold axis, respectively. The atom color code is the same as in Figure 1. The three disordered components are drawn with different colors (violet, green, and blue).

The most difficult part of the crystallographic analysis was modeling the inner core of **3** (details are provided in the Experimental Section). The two tripodal ligands in fact encapsulate a hexairon–oxo core that is massively disordered around the *D*_3_ symmetry site, meaning that the actual molecular symmetry is lower than *D*_3_. The final model encompasses a central Fe_5_(μ_5_-O) cluster with a distorted square-pyramidal geometry (trigonality parameter^61^ τ_5_ = 0.36 for the central O atom), orientationally disordered around the 3-fold axis (Figure 2). The Fe1 and Fe2 sites thus show partial occupancies of ^2^/_3_ and ^1^/_3_, respectively, while O1 has full occupancy and lies on the *D*_3_ symmetry site. On either side of the central core, two additional full-occupancy O atoms (O2 and O2^III^) are located on the 3-fold axis 2.796(7) Å from O1 (Figure 1).

The base of the pyramid is nonplanar, with an internal Fe1–Fe1^II^–Fe1^III^ angle of 84.85(4)° and an internal Fe1^II^–Fe1–Fe1^IV^ angle of 93.065(13)°. The length of the minor edge is 2.629(2) Å, suggesting a possible M–M bond within the Fe1, Fe1^II^ and Fe1^III^, Fe1^IV^ pairs. This distance is in fact comparable with the mean Fe–Fe separation (2.64 Å) in a variety of carbonyl-containing polyiron complexes^62^ and shorter than the partial Fe–Fe bonds (2.70 Å) in iron–sulfur clusters such as Roussin’s black salt.^63^ On the contrary, the Fe1–Fe1^IV^ and Fe1^II^–Fe1^III^ distances are much longer [3.028(3) Å] and indicate no M–M bond. Each Fe1 atom is five-coordinate with two pyridine-type nitrogen atoms (N_py_), one amido-type nitrogen atom (N_am_), O1, and O2 as ligands (Fe1–N, 2.129–2.151 Å; Fe1–O, 1.997–2.133 Å).

The apical site of the Fe_5_(μ_5_-O) pyramid is occupied by Fe2, whose distances to the basal metal sites are in the range of 3.232–3.451 Å. Fe2 is also five-coordinate with two pairs of symmetry-equivalent N_am_ and N_py_ atoms, and O1 as donors [Fe2–N_am_, 2.019(5) Å; Fe2–N_py_, 2.331(5) Å; Fe2–O1, 1.931(3) Å]. The rough square-pyramidal geometry has Fe2 lying ∼0.86 Å from the mean plane defined by its four basal N donors.

The central O^2–^ ion (O1) is found 0.75 Å from the mean plane defined by the four Fe1 ions. As mentioned above, it forms short contacts with the four Fe1 atoms [2.1327(13) Å] and an even shorter one with Fe2 [1.931(3) Å]. Similar square-pyramidal Fe_5_(μ_5_-O) units were previously observed in compounds [Fe_5_(μ_5_-O)(μ-OEt)_8_(OEt)_5_]^30^ (**5**) and [Fe_5_(μ_5_-O)(μ-O^*i*^Pr)_8_Cl_5_]^29^ (**6**). The cores of **5** and **6** are almost identical to each other, with square-like basal planes (internal Fe–Fe–Fe angles of 89.7–90.3°) and Fe–Fe separations of >3 Å, indicating no Fe–Fe bonds. Therefore, to the best of our knowledge, **3** is the first example of a complex with a distorted square-pyramidal Fe_5_(μ_5_-O) core and potential M–M bonds.

Fe3 is located in the coordination pocket of the tren-like moiety, ∼0.37 Å off the 3-fold axis, and is consequently disordered over three positions. Additionally, it is only half-occupied, affording an average number of six Fe centers per molecule. This is in accordance with EA and with the clear observation of a residual electron density peak attributable to the H(N2) hydrogen atom in Δ*F* maps (see the Experimental Section). Most likely, then, a single tren-like pocket per molecule (N1,N2,N2^I^,N2^II^ in Figure 1) undergoes deprotonation and hosts a metal ion, while the remaining one (N1^III^,N2^III^,N2^IV^,N2^V^ in Figure 1) retains its secondary NH groups and is unmetalated. Alternative scenarios cannot be excluded, like the occurrence of penta- and heptairon species mixed together in a 1:1 proportion in the crystal lattice. Clearly, X-ray diffraction and EA cannot distinguish between these limiting situations and all of the intermediate possibilities. Ionic species containing from five to seven metal centers were indeed detected by ESI-MS (see above), but signals from hexairon species were by far the most intense. This observation suggests that Fe_6_ complexes are dominant in both powder and crystalline samples, under the reasonable assumptions that no metal ion redistribution occurs in solution and that Fe_5_, Fe_6_, and Fe_7_ species are equally as easily ionized and fly equally well in the ESI-MS spectrometer.

The tren-like portion of the structure is different from that found in complexes of triply deprotonated H_3_py_3_tren, the shorter congener of H_6_L.^27^ In these complexes, the three secondary N atoms (N_am_) and the tertiary N donor of py_3_tren^3–^ simultaneously coordinate to a metal ion lying on the idealized 3-fold axis of the ligand. In **3**, the bulky iron–oxo core increases the divergence among the ligand’s three branches, affording larger distances between N_am_ atoms (3.93 Å vs 3.2–3.4 Å) and precluding their simultaneous coordination to Fe3. As a result, Fe3 has four short coordination bonds with O2, N1, and two N_am_ donors [1.851(8), 2.162(10), 2.180(12), and 2.129(11) Å, respectively] and a much longer contact with a third N_am_ atom [2.679(8) Å]. Considering that the ligand’s geometry is averaged over metalated and empty tren-like pockets, these bond distances may not accurately reflect the actual coordination geometry, as confirmed by the distinctly prolate displacement ellipsoid of N2 (Figure S5).

An intriguing structural feature is that, in spite of the severely disordered core, O2 is found exactly on the 3-fold axis within experimental resolution and has no abnormal displacement ellipsoid. Its protonation state could not be directly determined by inspection of Δ*F* maps but was inferred using BVS calculations and DFT studies (see below). Worth noting is also the acute Fe1–O2–Fe1^II^ angle of 82.3(2)°, which can be explained by the short Fe–Fe separation. A similar coordination geometry was recently observed in two isomers of hydroxo-bridged triosmium complex [Os_3_(CO)_8_(μ-OH)(μ-H)(μ-dppm)] [**7**, where dppm is bis(diphenylphosphino)methane].^64^ In complex **7**, an OH^–^ ion coordinates two Os atoms engaged in a M–M bond (Os–Os, 2.770–2.790 Å) and the Os–O–Os angle is as small as 80.4–81.1°.

### Bond Valence Sum Calculations

The massive disorder effects described in the previous section may easily lead to inaccurate bond lengths. Nevertheless, we attempted to validate the proposed structural model using BVS calculations (Table S2).^65^ The observed bond lengths support the assignment of the central oxygen (O1) as a μ_5_-O^2–^ ligand (BVSs of 1.95 and 2.08 for Fe^2+^ and Fe^3+^ parameters, respectively). The two identical Fe1–O2 distances instead afford BVSs of 0.98 and 1.05 for O2 and are thus suggestive of a μ-OH^–^ group when the neighboring tren-like pocket is unmetalated. What makes this hypothesis more realistic is the fact that O2 is surrounded by three N_py_ atoms almost exactly coplanar with it. The arrangement of these N_py_ atoms and the N_py_–O2 distance of 2.979(5) Å would favor an OH···N_py_ hydrogen bond of moderate strength (according to the classification of Jeffrey), as shown by the dashed lines in Figure 1.^66,67^ Addition of the Fe3–O2 contribution leads to BVSs of 1.71 and 1.83 for O2, which is suggestive of a μ_3_-O^2–^ group when the neighboring tren-like pocket is metalated. These protonation states were confirmed by DFT calculations (see below). Because no counterions were detected in the X-ray diffraction analysis, and conductivity measurements indicate nonelectrolyte behavior (see the next section), we formulate the compound as [Fe_6_O_2_(OH)(H_3_L)L] (**3**), which requires mixed valency with nominally four Fe^II^ ions and two Fe^III^ ions (average oxidation state of +2.33).

Calculations on Fe1, Fe2, and Fe3 with standard BVS parameters yield BVSs of 1.90/2.14, 2.01/2.30, and 1.84/2.09, respectively (Table S2). As a possible refinement, high-quality CSD data for HS Fe^2+^ complexes with dpa^–^ or tpda^2–^ ligands were also used to determine a specialized *R*_0_ parameter for Fe^2+^–N^3–^ bonds with N_py_ and N_am_ donors (Table S2 and Figure S6). The calculated BVS values are ∼0.1 valence units higher than the previous set based on standard Fe^2+^ parameters (Table S2). However, BVS calculations provide no clear-cut indication of valence distribution, presumably because of disorder effects and bond length inaccuracy.

### Electrochemistry

**Figure 3:**
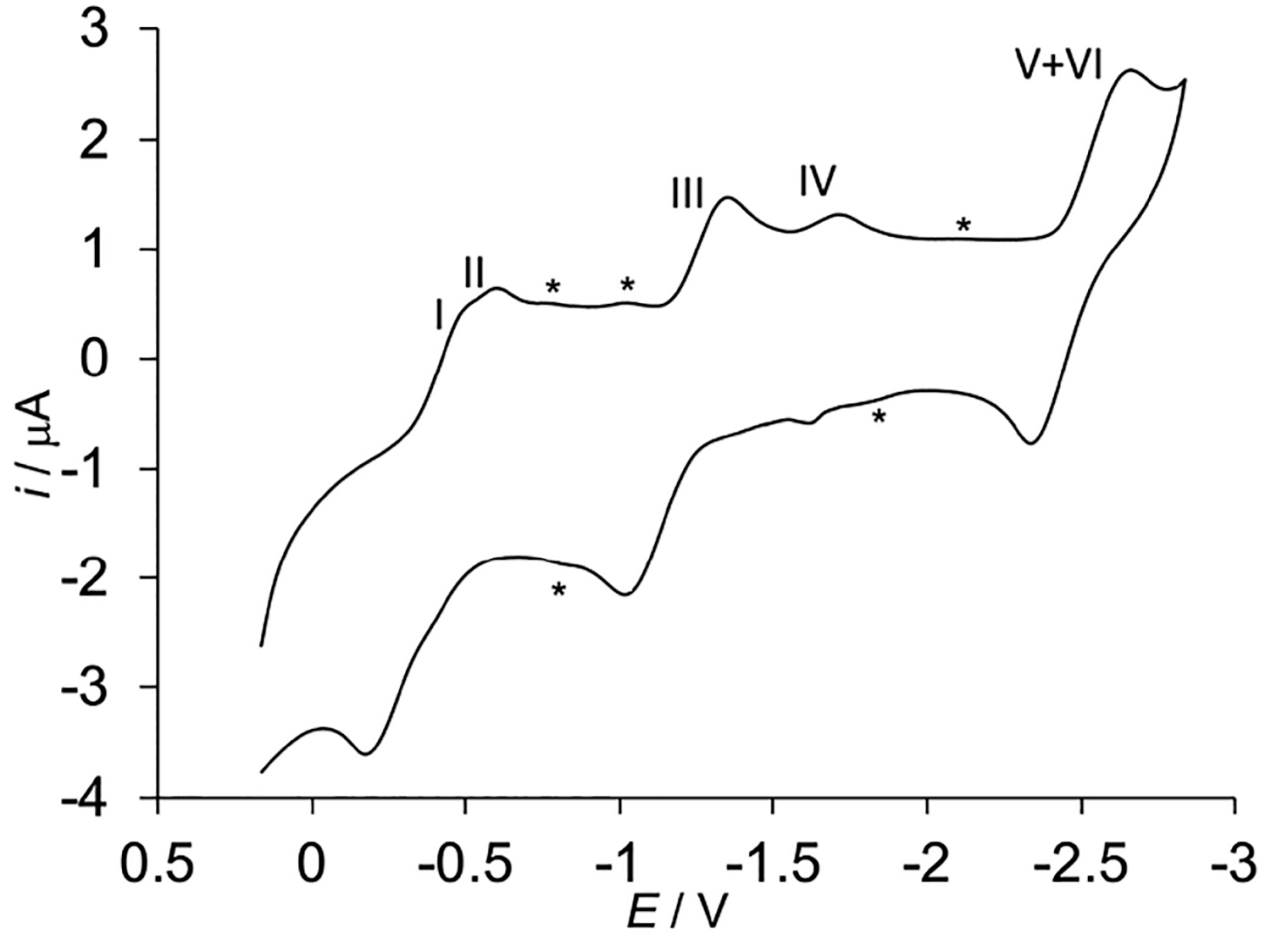
Cyclic voltammogram of **3** (0.25 mM in thf) at −10 °C. GC working electrode, 0.05 M TBATFB as the base electrolyte, scan rate of 0.05 V s^–1^, Fc^+^/Fc reference electrode. The signals marked with an asterisk have very low peak currents and confidently arise from minor additional species.

The CV curve for **3** in thf is shown in Figure 3. It presents five consecutive major reduction peaks with the corresponding oxidation counterparts (hereafter indicated as signals I–IV and V+VI at decreasing potential values). The peak-to-peak separation varies for the different signal couples, but in all cases, it increases with scan rate (*v*). The cathodic peak currents are proportional to the square root of *v*. This means that the electrochemistry of **3** consists of five quasi-reversible, diffusion-controlled redox processes. Other minor signals marked with an asterisk in Figure 3 have much lower peak currents and may arise from trace amounts of additional species. Hereafter, these will not be considered any further. The major signals are paired into three groups of redox couples, partially or completely overlapped. The similar total currents of these groups suggest that signals I–IV and V+VI are due to the same species holding several redox centers (Fe^III^/Fe^II^) and undergoing successive reduction/oxidation processes. The first group of signals consists of two distinct redox couples (signals I and II) that partially overlap. The second group also comprises two redox couples that, however, are better resolved. Signal III features an intense anodic current, which is approximately the sum of the cathodic currents of signals III and IV. The anodic peak of signal IV is rather low, but it grows at the detriment of anodic signal III with an increase in *v*. This suggests that the corresponding iron center changes its coordination environment upon reduction and that the reduced species reoxidizes at a potential very similar to that of signal III. In general, the cyclic voltammograms of polynuclear complexes contain signals from each redox center. The corresponding currents, however, can be affected (even deeply) by the stability of the redox state that is formed in the previous ET step.^68−70^ We performed CV measurements at a high potential scan rate and restricted the explored potential range to minimize the time required to complete CV. This would limit the degradation of the redox form generated in reduction step III. In fact, with an increase in the scan rate from 50 to 500 mV/s, we observe a remarkable increase in the intensity of signal IV and of its oxidation counterpart. At the same time, the intensity of the oxidation counterpart of signal III decreases (Figure S7) while the *E*°′ values remain almost unchanged. At both scan rates, the sum of the cathodic currents equals that of the anodic ones, but they are differently distributed between signals III and IV. This confirms that the two signals refer to subsequent processes of the same species. The more negative signal (signal V+VI) is very intense and characterized by a cathodic peak current remarkably higher than those of the other cathodic signals, while the anodic current is similar to that of signal III and to the sum of the anodic currents of signals I and II. Overall, the CV response supports our proposal that hexairon complexes are largely dominant in **3**, with penta- and heptairon complexes as possible minority components (see ESI-MS results). The formal potentials *E*°′ and the standard heterogeneous ET rate constants *k*_ET_ associated with the redox processes are listed in Table 2.

**Table 2:** Electrochemical Data from CV for the Consecutive ET Processes of **3**^a^

	*E*°′ (V)^b^	Δ*E*°′ (V)^c^	*K*_c_^d^	*k*_ET_ (cm s^–1^)^e^
*E*(I)	–0.314			0.0028
		0.157	9.01 × 10^2^	
*E*(II)	–0.471			0.0033
		0.706	1.93 × 10^13^	
*E*(III)	–1.177			0.0027
		0.457	3.98 × 10^8^	
*E*(IV)	–1.634			0.0046
		0.871	2.45 × 10^16^	
*E*(V+VI)	–2.505			0.0026

a Experimental conditions: 0.25 mM in thf, −10 °C, GC working electrode, 0.05 M TBATFB as the base electrolyte, scan rate of 0.05 V s^–1^. The average errors on *E*°′, Δ*E*°′, *K*_c_, and *k*_ET_ are ±0.002 V, ±0.004 V, ±16%, and ±8%, respectively.

b Formal reduction potential (referenced to the Fc^+^/Fc redox couple).

c Separation between *E*°′ values of consecutive ET processes.

d Comproportionation constant.

e Heterogeneous ET rate constant.

Complex **3** shows an unusual redox behavior because its ET processes span a very large potential window (∼2.2 V). The occurrence in the cyclic voltammogram of quasi-reversible processes that appear to be coupled in pairs suggests the presence of electronic interactions between the iron ions and minor structural reorganization upon changing the redox state, except for signal IV whose reduced form is unstable.^71^ The separations between *E*°′ values of consecutive ET processes (Δ*E*°′) are mostly large, and only signals I and II are partially overlapped (Table 2). This possibly reflects the stabilization energy imparted to the complex by electron delocalization.^72,73^ The Δ*E*°′ values mostly correspond to very large comproportionation constants {*K*_c_ = exp[*nF*(*E*°_1_ – *E*°_2_)/(*RT*)]}^74,75^ ranging between 10^8^ and 10^16^, and only the *K*_c_ value related to signals I and II is quite small (9.01 × 10^2^). Large values of *K*_c_ indicate a high thermodynamic stability of the redox-active forms of the complex toward disproportionation (Table 2).^76^ The negative *E*°′ values, although distributed over a wide range, are comparable to those found for other related iron complexes.^77,78^

The *k*_ET_ values for the observed ET processes are low and rather similar (Table 2). This could be related to a high reorganization energy λ, already observed for other mixed valence complexes.^79^

The molar conductivity measured between −23 and 2 °C on a 0.25 mM solution in thf is on the order of 0.1 Ω^–1^ cm^2^ mol^–1^ and depends only slightly on temperature (Table S3). Being much lower than found for ionic complexes in the same solvent^80^ or in other organic solvents,^81^ it suggests that **3** is a nonelectrolyte in thf.

### Mössbauer Spectroscopy

**Figure 4:**
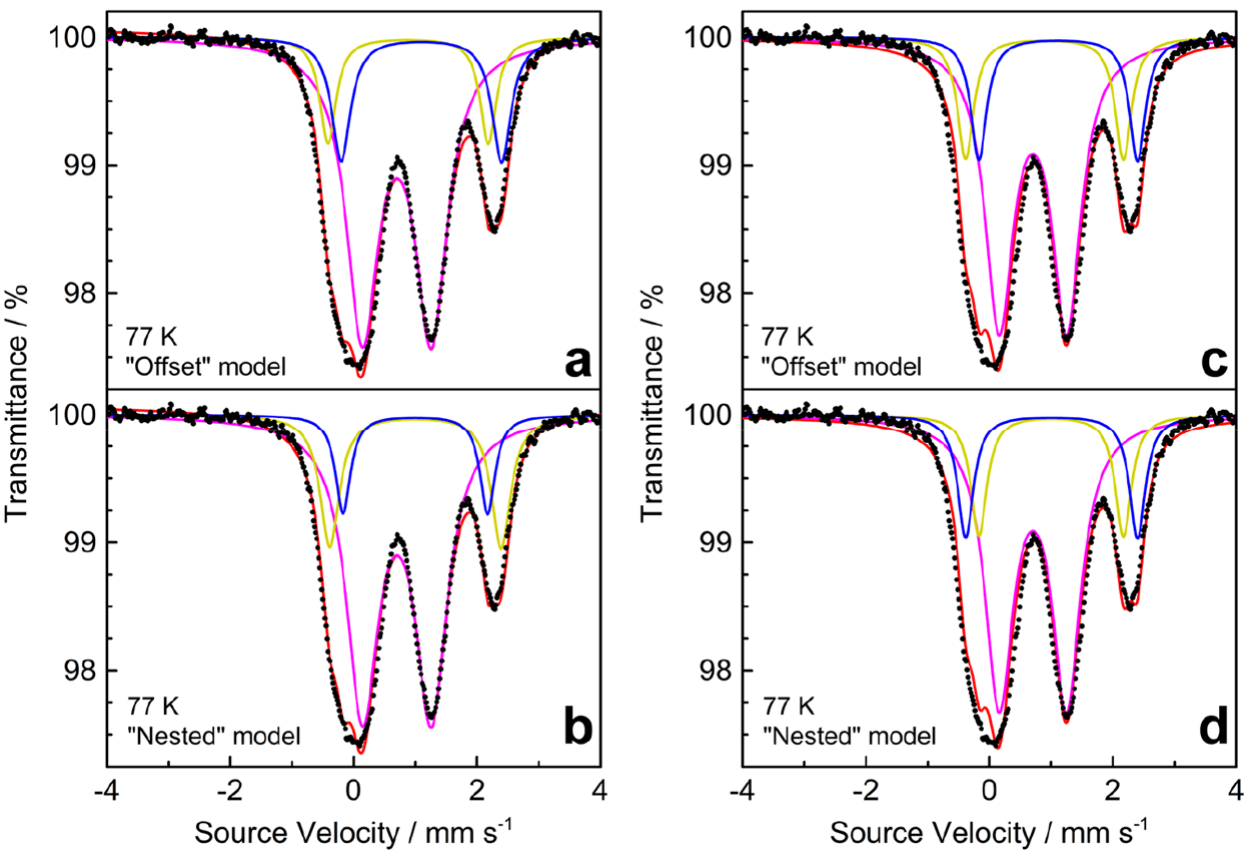
Mössbauer spectrum of **3** at 77 K (solid dots) with accompanying (a) **Fit1**, (b) **Fit2**, (c) **Fit3**, and (d) **Fit4** as red lines. The red traces are additionally divided into three quadrupole doublets (purple, yellow, and blue lines). The three contributions in panels c and d are constrained to account for 66.7%, 16.7%, and 16.7%, respectively, of the total Fe content.

To assist in assigning oxidation states and spin states to the Fe ions in **3**, Mössbauer spectra were recorded on a polycrystalline sample at 77 K (Figure 4) and 10 K (Figure S8). In the following analysis, as in the discussion of magnetic data, the contributions from minority species possibly co-crystallized with **3** are neglected. The 10 K spectrum appears to be broadened by magnetic relaxation effects and will not be discussed further. Thus, we focus our discussion on the spectrum recorded at 77 K, which contains three major features at 0.07, 1.27, and 2.27 mm s^–1^. These features are much broader than the typical Mössbauer line width for a single Fe site (Γ_fwhm_ ∼ 0.3 mm s^–1^), indicating that multiple Fe sites contribute to each of them. This is not surprising, because crystallographic symmetry already requires three different Fe centers in a 4:1:1 ratio, namely the set of basal sites (Fe1, Fe1^II^, Fe1^III^, and Fe1^IV^), apical site Fe2, and peripheral site Fe3 located in the tren-like pocket (Figure 1). Several models for the 77 K spectrum were investigated using three unique quadrupole doublets with adjustable relative areas. Here, we focus on fits in which the spectral peak at 2.27 mm s^–1^ is modeled with two overlapping subspectra (**Fit1** and **Fit2** in Figure 4), though models with a single quadrupole doublet (**FitS1** and **FitS2** in Figure S9) were also considered. In either case, there are two nearly identical overall fits depending on whether the two quadrupole doublets that more extensively overlap are offset (**Fit1** and **FitS1**) or nested (**Fit2** and **FitS2**). Unfortunately, it is not possible to determine whether the offset or the nested models are more correct.

In **Fit1** and **Fit2** (Figure 4), the two dominant lines are reproduced by a single, broad quadrupole doublet that accounts for 69% of the total iron content. The best-fit parameters (δ = 0.70 mm s^–1^, and Δ*E*_Q_ = 1.11–1.12 mm s^–1^) are consistent with mixed HS iron(II)/iron(III) character.^82^ However, the large line width of this signal (Γ_fwhm_ ∼ 0.63 mm s^–1^) suggests contributions from multiple Fe sites. Efforts to use more than one quadrupole doublet afforded multiple equally stable solutions, and no new information was gained from such overparameterized fits. Two unique quadrupole doublets with larger δ and Δ*E*_Q_ values and a more normal line width (∼0.3 mm s^–1^) are needed to fit the remaining features of the spectrum, namely, the low-velocity edge of the main signal and the smaller high-velocity feature. An offset model (**Fit1**) yields different isomer shifts for these doublets (0.88 and 1.10 mm s^–1^), but the same quadrupole splitting (2.60 mm s^–1^). **Fit2** has nested doublets with the same isomer shift (1.00 mm s^–1^) and different quadrupole splittings (2.78 and 2.34 mm s^–1^).

In both cases, the Mössbauer parameters are typical for HS iron(II) ions.^82^ The integrated intensity of each of these doublets corresponds to 12–19% of the total area of the spectrum. Overall, the relative areas of the three subspectra in **Fit1** and **Fit2** deviate only slightly from the 4:1:1 pattern (66.7:16.7:16.7) of Fe sites in the crystal structure. As a consequence, two additional models (**Fit3** and **Fit4** in Figure 4) were considered with a fixed 4:1:1 population. The best-fit parameters so obtained are fairly similar to those in the unconstrained models. As in **Fit1** and **Fit2**, the two major features may be fitted to a broad quadrupole doublet with δ = 0.71 mm s^–1^ and Δ*E*_Q_ = 1.09 mm s^–1^, accounting for ^2^/_3_ of the total iron content. The remainder of the spectrum is fitted to two quadrupole doublets with populations fixed at ^1^/_6_ that are either offset or nested. The offset model (**Fit3**) has different isomer shifts (0.89 and 1.11 mm s^–1^) and similar quadrupole splittings (2.56–2.57 mm s^–1^). The nested model (**Fit4**) has similar isomer shifts (1.00–1.01 mm s^–1^) and distinct quadrupole splittings (2.79 and 2.34 mm s^–1^).

Comparing the extracted Mössbauer parameters, which are listed in Table S4, to those of known, structurally related compounds is difficult. Most of the Fe site geometries in **3** are highly unique and have little direct structural precedent. The Fe site located within the tren-like pocket (Fe3) is reminiscent of similar sites in dinuclear compounds [Fe_2_Cl(py_3_tren)] (**8**) and [FeMnCl(py_3_tren)] (**9**).^27^ However, the Mössbauer parameters in these complexes may be strongly affected by metal–metal bonding. For instance, the isomer shifts in **8** and **9** (0.46–0.48 mm s^–1^) are unusually low and fall outside the expected range for HS iron(II), while the quadrupole splittings (1.31–1.69 mm s^–1^) lie at the lower edge of the range typical for HS iron(II) ions.^82^

In summary, the Mössbauer spectrum of **3** is complex, as expected for the large number of independent Fe sites. We have attempted to find a physically meaningful fit with the fewest independent parameters. Stable fits with an approximate 4:1:1 ratio of Fe signals indicate the presence of two unique Fe sites with large quadrupole splitting values and clear HS iron(II) character. The Mössbauer parameters for the remainder of the Fe sites are instead suggestive of a mixed HS iron(II)/iron(III) character. These results are thus consistent with the proposed occurrence of four HS iron(II) ions and two HS iron(III) ions in the structure. This assignment is reasonable considering that strong delocalization of a mixed valent system causes both isomer shifts and quadrupole splittings to be observed in the range between what is expected for HS iron(II) and iron(III).

### Magnetic Susceptibility Measurements

**Figure 5:**
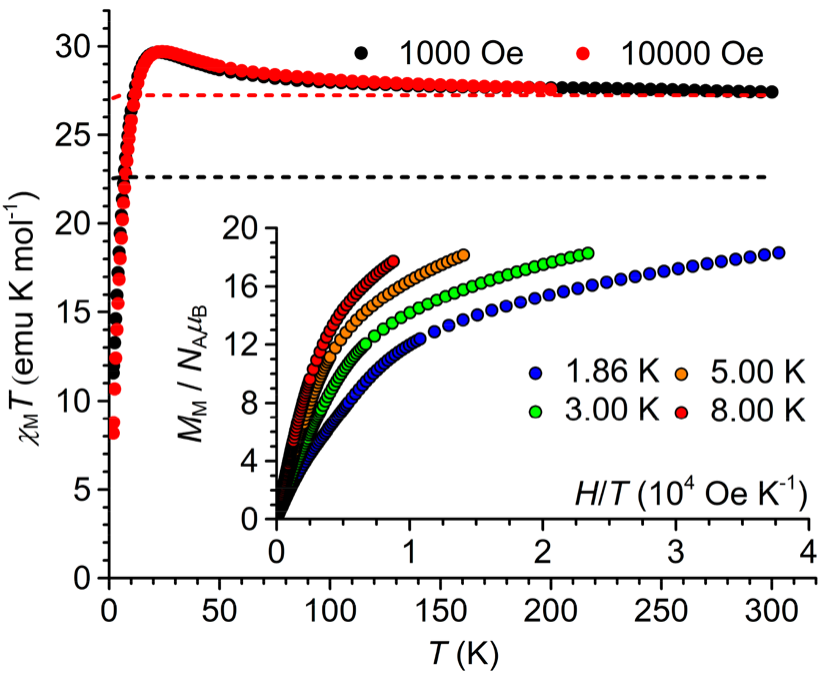
Plot of χ_M_*T* vs *T* for **3** recorded at 1 and 10 kOe. The dashed curves are the expected magnetic responses for one *S* = 4, two *S* = 2, and two *S* = ^5^/_2_ uncorrelated spins (red) and for four *S* = 2 and two *S* = ^5^/_2_ uncorrelated spins (black), taking *g* = 2.15 for *S* = 4 and *S* = 2 and *g* = 2.00 for *S* = ^5^/_2_. The inset shows reduced magnetization curves at 1.86 K (blue), 3.00 K (green), 5.00 K (orange), and 8.00 K (red).

The variable-temperature molar magnetic susceptibility (χ_M_) of **3** is shown in the main panel of Figure 5 as a plot of χ_M_*T* vs *T*, while the isothermal molar magnetization (*M*_M_) measured at low temperature is plotted in the inset as a function of *H*/*T*. A χ_M_*T* value of 27.4 emu K mol^–1^ is found at room temperature, corresponding to a magnetic moment of 14.8 μ_B_ per molecule. As temperature is lowered, the χ_M_*T* product at 1 kOe gradually increases, reaching a maximum of 29.7 emu K mol^–1^ at 22 K before decreasing precipitously to 11.6 emu K mol^–1^ at 1.85 K. The initial increase in χ_M_*T* upon cooling from room temperature suggests that ferromagnetic interactions are operative in the compound, while the final low-temperature decrease is indicative of either antiferromagnetic interactions, magnetic anisotropy, or a combination of the two effects.^17^ Using the proposed oxidation state assignment of four HS iron(II) ions (*S* = 2) and two HS iron(III) ions (*S* = ^5^/_2_), the high-temperature value expected for χ_M_*T* is 20.8 emu K mol^–1^ with a *g* of 2.00 for all metal ions, or 22.6 emu K mol^–1^ with *g* values of 2.15 and 2.00 for Fe^2+^ and Fe^3+^ ions, respectively (Figure 5, black dashed line). These values are significantly lower than that observed at room temperature (27.4 emu K mol^–1^) and suggest the presence of a strong ferromagnetic interaction influencing the room-temperature χ_M_*T* value.

Considering the relatively short Fe1–Fe1^II^ distance of 2.63 Å (equivalent to the Fe1^III^–Fe1^IV^ distance), the room-temperature χ_M_*T* value could be explained by the presence of a ferromagnetically coupled iron(II)–iron(II) partially bonded dimer. A full bond between two HS iron(II) ions at a distance of 2.46 Å was indeed described to afford a thermally persistent *S* = 4 ground state.^83,84^ If **3** is considered to contain such an *S* = 4 contribution, along with two isolated HS iron(II) centers and two isolated HS iron(III) centers, then the expected room-temperature χ_M_*T* value is 24.8 emu K mol^–1^ with a *g* of 2.00 for all metal ions, or 27.3 emu K mol^–1^ with *g* values of 2.15 and 2.00 for Fe^2+^ and Fe^3+^ ions, respectively (Figure 5, red dashed line). These calculated data provide a much better match for the experimental value of 27.4 emu K mol^–1^ (Figure 5). The existence of such a strongly ferromagnetically coupled iron(II) pair within the hexairon–oxo core of **3** was supported by DFT calculations described in the next section. The overall shape of the χ_M_*T* vs *T* curve suggests that the remaining magnetic interactions in **3** are weak, as confirmed by the estimate provided in the Supporting Information (Figure S10).

The molar magnetization data at 1.86 K (Figure 5, inset) increase to 18.3 *N*_A_μ_B_ at the highest applied field without saturating. This suggests a ground electronic state with at least 18 unpaired electrons, which would afford a saturation magnetization of 18.9 *N*_A_μ_B_ assuming an average *g* of 2.10.

To probe any slow magnetic relaxation, the ac magnetic susceptibility of **3** was measured at low temperatures. Both in-phase (χ′) and out-of-phase (χ″) components depend on the frequency (ν) in zero dc field, indicating that the compound is a single-molecule magnet (Figure S11). The fitting of the plots of χ′ vs ν and χ″ vs ν leads to a relaxation time (τ) that follows an activated behavior between 1.9 and 2.8 K with an energy barrier (*U*_eff_/*k*_B_) of 49(2) K and a pre-exponential factor (τ_0_) of 4(2) × 10^–10^ s (Figures S11–S15). Considering the very small temperature domain that is available for studying the relaxation, it is difficult to be absolutely sure that the relaxation follows an Orbach process. Nevertheless, the weak field dependence of the relaxation time at 2 K up to 20 kOe supports this interpretation (Figures S13–S15).

### Density Functional Theory Calculations

**Figure 6:**
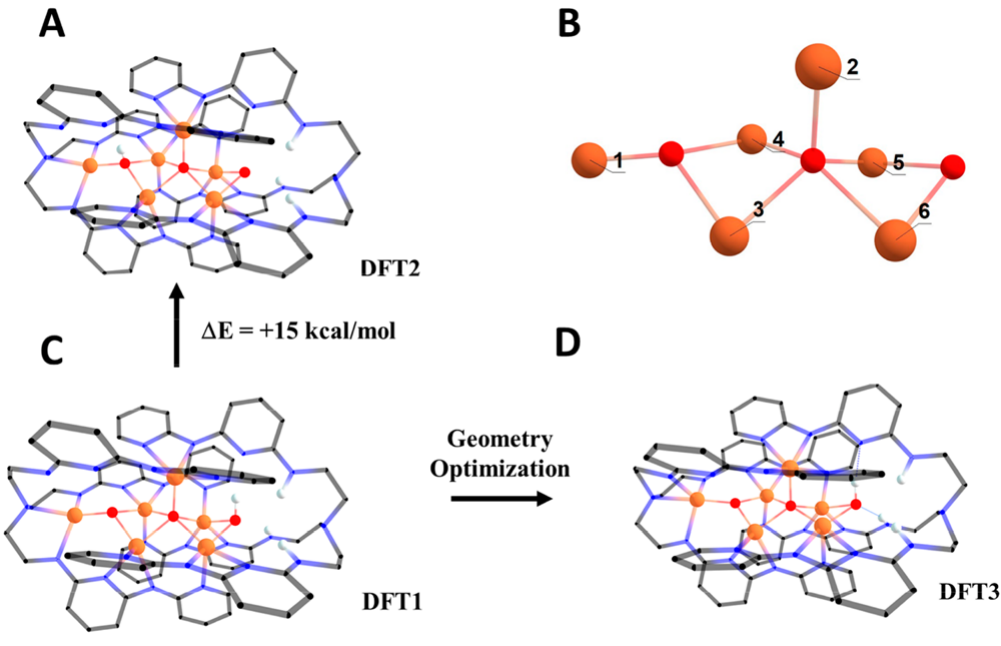
(A and C) Models **DFT2** and **DFT1**, respectively, with hydrogen atom positions optimized. (B) Labeling scheme for metal ion sites. (D) Model **DFT3** with optimized positions for all atoms. In these diagrams, all hydrogen atoms bound to carbon have been omitted for the sake of clarity and the organic ligand is represented by a wireframe model. See the Supporting Information for full structural descriptions of **DFT1**–**DFT4**.

To interrogate the protonation state of oxygen atoms inferred from crystallographic data and BVS values, DFT calculations were employed. The crystallographic coordinates of **3** were used as a starting point for the construction of a pair of computational models containing two HS iron(III) ions and four HS iron(II) ions, but different proton distributions on the three O atoms (O2, O1, and O2^III^): (oxo, oxo, hydroxo) in model **DFT1** and (hydroxo, oxo, oxo) in model **DFT2** (see Table 3 and Figure 6). Notice that the Fe site labeling in Figure 6 does not follow the crystallographic scheme of Figure 1: 1 is the metal site within the tren-like pocket, while 2 and 3–6 are the apical and basal metal sites in the pentairon core, respectively. The HS states for each structure were used to avoid convergence problems anticipated for spin-coupled states, and all of the hydrogen atom positions were optimized. The electronic energy of **DFT2** calculated using the B3LYP functional is 15 kcal mol^–1^ higher than that of **DFT1**, showing that the (oxo, oxo, hydroxo) isomer is energetically more stable than the (hydroxo, oxo, oxo) isomer. Because of its higher electronic energy, **DFT2** was not considered in any of the following analyses, nor did we consider this proton distribution in our full geometry optimizations.

**Table 3:** Computational Details for Models **DFT1**–**DFT4**

model	metal content and formal oxidation states	*S*	oxygen ligands (O2, O1, O2^III^)	(*i*, *j*); *J*_*i*__,*j*_ (cm^–1^)^a^	functional
**DFT1**	Fe^II^_4_Fe^III^_2_	13	oxo, oxo, hydroxo	–	B3LYP
**DFT2**	Fe^II^_4_Fe^III^_2_	13	hydroxo, oxo, oxo	–	B3LYP
**DFT3**	Fe^II^_4_Fe^III^_2_	13	oxo, oxo, hydroxo	–	BP86
**DFT4**	Fe^II^_2_Ga^III^_2_Zn^II^_2_^b^	4	oxo, oxo, hydroxo	(5, 6); 67	BP86

a The Heisenberg Hamiltonian is defined as in PHI,^85^ i.e., –2*J*_*i*__,*j*_**Ŝ**_*i*_·**Ŝ**_*j*_.

b The model encompasses Fe at sites 5 and 6, Ga at sites 1 and 3, and Zn at the remaining metal sites.

All atomic positions of **DFT1** were successfully optimized at the BP86 level of theory to give model **DFT3**. To evaluate the structural match of **DFT3** with X-ray diffraction data, we calculated the root-mean-square deviation (RMSD) of atomic positions from the crystallographic coordinates. This structural overlay considered the Fe, O, and N atoms only and gave a RMSD of 0.26 Å. Despite the low RMSD, **DFT3** does exhibit some distortion from the crystallographic coordinates, most notably in the contraction of the Fe3–N2 distance (see Figure 1 and Table S5). The shortest Fe–Fe separation (2.71 Å) is found between sites 5 and 6, i.e., along the OH-bridged base edge of the Fe_5_(μ_5_-O) square pyramid; on the contrary, the distance between sites 3 and 4 along the opposite base edge is 2.84 Å and the length of two remaining base edges is 2.95–3.07 Å.

The valence distribution in **DFT3** was checked by BVS calculations^65,86^ and spin population analysis. BVS values (Table 4) indicate clearly that sites 5 and 6 have the largest iron(II) character among the six metal sites. The spin populations support this assignment, indicating that sites 5 and 6 have the lowest spin population of the Fe atoms, consistent with HS iron(II) vs HS iron(III) (see Table S6 for a full analysis). This optimized structure was subsequently used in a single-point calculation to predict the Mössbauer parameters (δ and Δ*E*_Q_) from the calculated wave function. It has been noted that quadrupole splittings for HS iron(II) and iron(III) are generally underestimated by GGA functionals such as BP86.^87^ Indeed, the calculated Δ*E*_Q_ values (Table S7) do not help us to discriminate among the possible Mössbauer models. Mössbauer isomer shifts for **DFT3**, calculated using the methods outlined by Neese and co-workers,^88^ are listed in Table 4. The calculated isomer shift for site 1 (0.58 mm s^–1^) is at the high end of the HS iron(III) range, but at the low end for the HS iron(II) range.^82^ This intermediate δ value may indicate some delocalization of the mixed valency. Notably, the average of the four lowest calculated isomer shifts is 0.71 mm s^–1^, while the other two calculated δ values are 0.86 and 0.97 mm s^–1^. Considering the expected error associated with the prediction of Mössbauer isomer shifts using BP86 (∼0.1 mm s^–1^), these three values are in satisfactory agreement with the experimental values from **Fit3** and **Fit4** (0.71, 0.89–1.00, and 1.01–1.11 mm s^–1^). From these results, we can establish that Mössbauer parameters from model **DFT3** with four HS iron(II) ions and two HS iron(III) ions are consistent with the available experimental data. Furthermore, in combination with BVS and spin population analyses, they help in the assignment of a prevalent HS iron(II) character to metal sites 5 and 6. The remaining four metal sites (Fe^II^_2_Fe^III^_2_) display substantial valence delocalization. However, combined BVS values, spin population data, and predicted Mössbauer parameters suggest that iron(III) character decreases slightly in the following order: 1 > 3 > 4 > 2.

**Table 4:** Values of BVS and Isomer Shift (mm s^–1^) for the Six Metal Sites in **3** as Obtained from Model **DFT3**, and Experimental Isomer Shifts from **Fit3** and **Fit4**

metal site^a^	BVS(Fe^2+^)^b^	BVS(Fe^3+^)^b^	BVS(Fe^2+^)^c^	δ from **DFT3**	δ from **Fit3** and **Fit4**^d^
1	2.165	2.471	2.276	0.58	0.71 (4)
2	2.014	2.318	2.153	0.75	
3	2.141	2.417	2.249	0.76	
4	2.110	2.388	2.221	0.76	
5	1.932	2.190	2.037	0.86	0.89–1.00 (1)
6	1.785	2.021	1.880	0.97	1.01–1.11 (1)

a For the labeling of metal sites, see Figure 6.

b Using the standard set of Fe^2+^ or Fe^3+^ BVS parameters from file bvparm2020.cif available at https://www.iucr.org/resources/data/datasets/bond-valence-parameters and including only bond distances within 3.0 Å. See Table S2 for details.

c Using the specialized *R*_0_ value for HS Fe^2+^ complexes with N_py_ and N_am_ donors and including only bond distances within 3.0 Å. See Table S2 and Figure S6 for details.

d Numbers in parentheses are the relative areas of the three subspectra.

An extensive network of weak Fe–Fe bonding interactions is found in **DFT3**, as evidenced by Mayer bond order (MBO) values between 0.1 and 1 (Table S8).^89^ There are interactions (MBO = 0.14–0.45) between site 2 and two of the four Fe atoms that make up the roughly square basal plane of the pentairon core (sites 3–6). Several relatively strong interactions (MBO = 0.16–0.35) also occur between adjacent Fe atoms in the square base.

We have further utilized DFT methods to probe magnetic interactions in **3**. Specifically, we decided to test the hypothesis, suggested by the magnetic susceptibility data, that the compound contains a ferromagnetically coupled diiron(II) unit with a local *S* = 4 state that persists to room temperature. A full analysis of the spin ladder is impractical and would also be inaccurate. As an alternative, starting from optimized structure **DFT3**, we assigned sites 5 and 6 as iron(II) ions and magnetically isolated this metal pair by replacing all other metal ions with diamagnetic substitutes of similar ionic radii. Gallium(III) was placed in sites 1 and 3, in agreement with the available data indicating that these sites have the largest iron(III) character, and zinc(II) was placed in the remaining metal sites (Figure 6). We do not expect this method to be quantitatively accurate, but this calculation was performed to determine if it supported the hypothesis that a strong ferromagnetic interaction can occur between iron(II) ions in sites 5 and 6. We therefore performed broken-symmetry single-point calculations on the resulting model (**DFT4**) to predict exchange coupling parameter *J* between these two Fe sites under the Heisenberg Hamiltonian (Table 3). The results indeed indicate strong ferromagnetic exchange coupling, with a *J* value of 67 cm^–1^. The orbitals involved in this exchange interaction can be found in Figure S16. They indicate a range of interactions between each pair of magnetic orbitals including oxo/hydroxo-mediated superexchange interactions that are ferromagnetic due to the Fe–O–Fe angles being close to 90° (82° and 79° for the oxo and hydroxo groups, respectively). These angles are even closer to 90° in the crystal structure; therefore, we may consider calculated exchange parameter *J* to be a lower bound. The large, positive *J* for this diiron(II) unit may be contextualized within the class of known Fe_2_(μ-X) structures. Though many examples of oxo/hydroxo-bridged compounds are known, magnetostructural studies are exceedingly rare, being limited to a hydroxo/aquo-bridged diferrous compound reported by Rybak-Akimova and co-workers in 2005.^90^ Also relevant to this study are an Fe^II^_2_(μ-F)_2_ complex described by Que and co-workers^91^ and a macrocyclic compound reported by Gagné, Hendrickson, and co-workers that contains an Fe^II^_2_ unit bridged by aryloxy groups.^92^ In each of these examples, the Fe–Fe separations are >3.1 Å and the Fe–(μ-X)–Fe angles are all >90°, which results in small exchange coupling constants (−9.6 cm^–1^ < *J* < 1.2 cm^–1^). The shorter Fe–Fe distance and the acute Fe–O–Fe angles found in **DFT4** will lead to stronger antiferromagnetic interactions (via direct exchange) as well as stronger ferromagnetic interactions (via superexchange with Fe–O–Fe angles closer to 90°) in comparison to those of the known compounds. Altogether, the ferromagnetic interactions dominate and iron(II) sites 5 and 6 may be reasonably expected to be ferromagnetically coupled to give an *S* = 4 state. As shown in the Supporting Information, the large *J* value implies that thermal population of lower spin states remains minimal up to room temperature, consistent with the magnetic susceptibility data (Figure S10).

## Conclusion

With their arrays of spatially organized N donors, oligo-α-pyridylamides and related ligands offer a viable route to polymetallic complexes showing a linear structure and, in many cases, metal–metal bonding (EMACs). Among first-row metals, iron is of special interest because short Fe–Fe separations often produce strong ferromagnetic couplings and large room-temperature magnetic moments.^13,14^ However, nuclearity is often lower (and Fe–Fe distances are often longer) than expected from the ligand’s structure.^14,16,17,60,93^ Moreover, the high oxophilicity of Fe leads to the easy incorporation of adventitious oxygen atoms to yield iron–oxo cores.^59,60^

Our findings indicate that these tendencies can be further accompanied by partial oxidation of Fe. Attempts to access iron(II)-based EMACs were made by reacting organometallic precursor [Fe_2_(Mes)_4_] with a tripodal proligand (H_6_L) containing three oligo-α-pyridylamine branches linked to a tren-like aliphatic pocket. The structure of H_6_L, which contains 13 nitrogen donors of four different types, was designed to favor the assembly of iron(II) ions into closely spaced linear arrays with metal–metal bond distances. The reaction instead resulted in the buildup of an organic nanocage comprising two tripodal ligands with interdigitated branches and hosting a mixed valence hexairon–oxo core. The formula and structure of the compound, [Fe_6_O_2_(OH)(H_3_L)L] (**3**), were inferred by combined use of EA, ESI-MS, electrochemical studies, X-ray crystallography, BVS analysis, and DFT calculations. In spite of the trigonal symmetry of the organic envelope, **3** displays a remarkably unsymmetric iron–oxo core orientationally disordered around a crystallographic *D*_3_ position. The core was modeled as [Fe(μ_3_-O)Fe_5_(μ_5_-O)(μ-OH)]^9+^, with a central Fe_5_(μ_5_-O) square pyramid supported by two additional oxygen bridges and a sixth Fe center located in the tren-like pocket of L^6–^. Mössbauer spectra confirmed that **3** is a mixed valence Fe^II^_4_Fe^III^_2_ species, containing two predominantly HS Fe^II^ sites and a substantially valence-delocalized Fe^II^_2_Fe^III^_2_ unit. Using BVS analysis and DFT calculations, the two unique metal sites were identified as a pair of closely spaced hydroxo-bridged metals in the central Fe_5_(μ_5_-O) pyramid. Significantly, broken-symmetry DFT calculations anticipate strong ferromagnetic coupling within this pair to give a thermally persistent *S* = 4 state, which explains the large magnetic moment of **3** at room temperature. As a final point of interest, slow magnetic relaxation was detected in the compound even in zero static field, with the following Orbach activation parameters: *U*_eff_/*k*_B_ = 49(2) K, and τ_0_ = 4(2) × 10^–10^ s.

## Supporting Information Available

ESI-MS, UV–vis–NIR, and NMR spectra, additional crystallographic figures and tables, details of BVS calculations, additional electrochemical data, conductivity measurements, additional Mössbauer spectra, details of Mössbauer and magnetic analyses, ac magnetic data, and details of DFT calculations (PDF)
Cartesian atomic coordinates of models **DFT1**–**DFT4** (TXT)

## Footnotes

a We herein refer to the maximum symmetry allowed by chemical structure. The actual symmetry in the solid state is usually lower.
