# Supplementary material for: A Remarkably Unsymmetric Hexairon Core Embraced by Two High-Symmetry Tripodal Oligo-α-pyridylamido Ligands
Source: Inorg Chem. 2023 Jun 22;62(26):10171–84. doi: 10.1021/acs.inorgchem.3c00808 (PMC10324315; doi:10.1021/acs.inorgchem.3c00808)
Supplement: Supplementary file 1 — ic3c00808_si_001.pdf [file ic3c00808_si_001.pdf]

A remarkably unsymmetric hexairon core embraced by two high-symmetry tripodal oligo-α-pyridylamido ligands

Alessio Nicolini,[a] Trey C. Pankratz,[b] Marco Borsari,[a] Rodolphe Clérac,[c] Antonio Ranieri,[d] Mathieu Rouzières,[c] John F. Berry,[b,*] and Andrea Cornia[a,*]

[a]Department of Chemical and Geological Sciences, University of Modena and Reggio Emilia & INSTM, I-41125 Modena, Italy

[b]Department of Chemistry, University of Wisconsin-Madison, 1101 University Avenue, Madison, WI 53706, USA

[c]Univ. Bordeaux, CNRS, CRPP, UMR 5031, F-33600 Pessac, France

[d]Department of Life Sciences, University of Modena and Reggio Emilia, I-41125 Modena, Italy

SUPPORTING INFORMATION

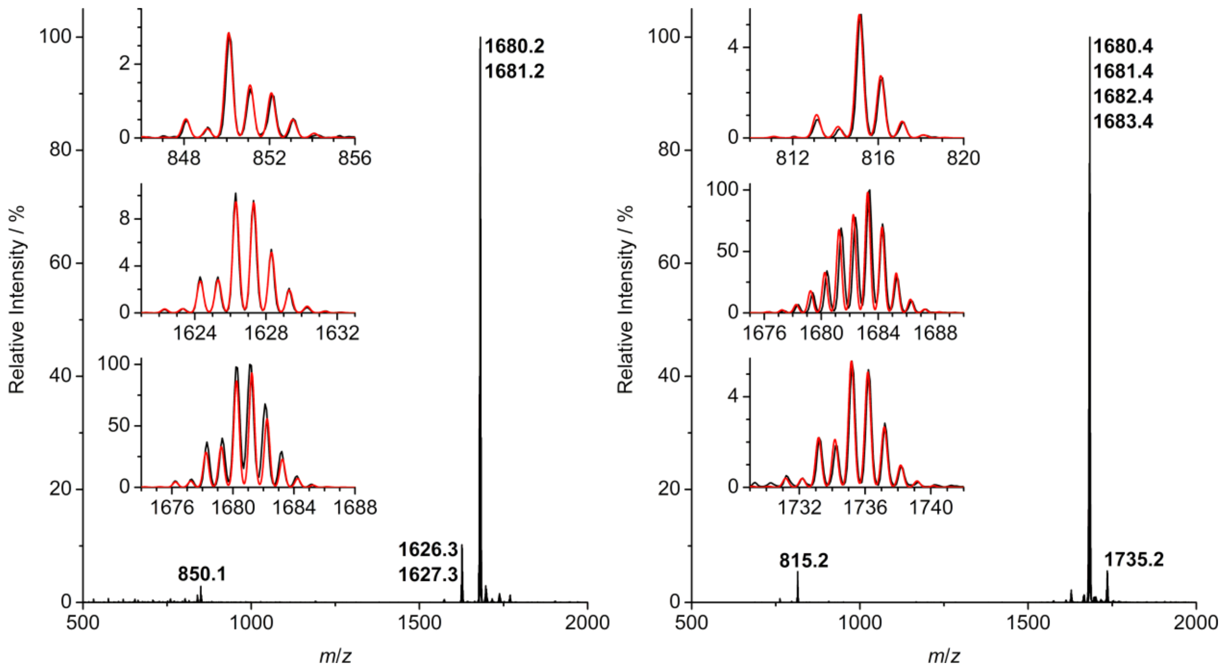

Figure S1. ESI-MS spectrum of **3**, in crystalline (left panel) and powder (right panel) form (direct infusion, thf, positive ion mode). The insets show the experimental (black line) and simulated (red line) isotopic patterns of the peaks, assigned as follows. Left panel: *m*/*z* = 850.1 ($[Fe_3ClL]^+$), 1626.3−1627.3 ($[Fe_5O_3H_4L_2]^+$ and $[Fe_5O_3H_5L_2]^+$, 0.9:0.1 ratio), 1680.2−1681.2 ($[Fe_6O_3H_2L_2]^+$ and $[Fe_6O_3H_3L_2]^+$, 0.8:0.2 ratio). Right panel: *m*/*z* = 815.2 ($[Fe_3L]^+$), 1680.4−1681.4−1682.4−1683.4 ($[Fe_6O_3H_2L_2]^+$, $[Fe_6O_3H_3L_2]^+$, $[Fe_6O_3H_4L_2]^+$, and $[Fe_6O_3H_5L_2]^+$, 0.08:0.24:0.24:0.44 ratio) and 1735.2 ($[Fe_7O_3HL_2]^+$).

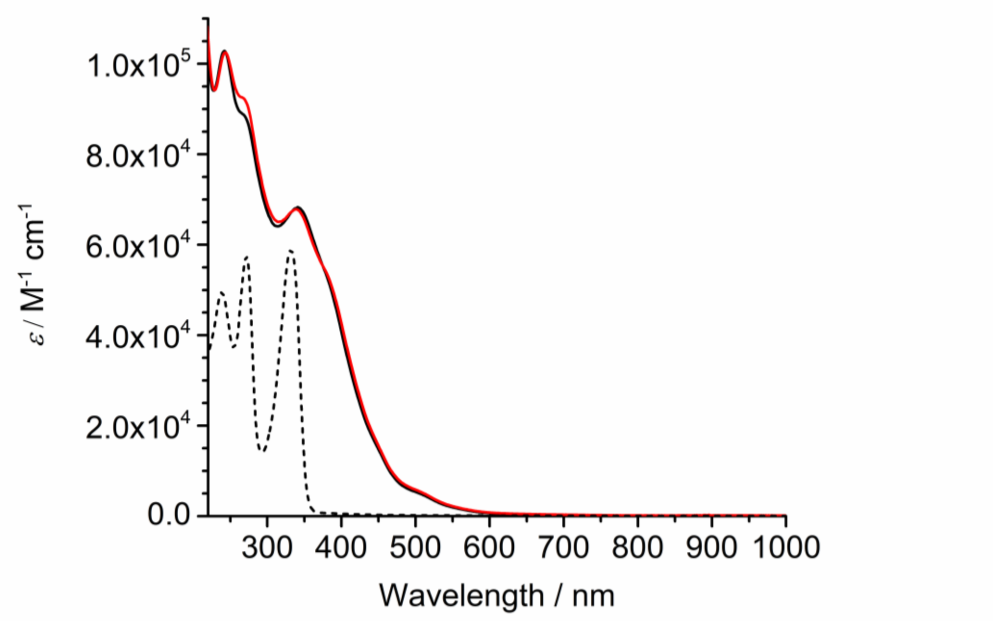

Figure S2. UV-Vis-NIR spectra of thf solutions of the H_6L proligand (dashed black line) and of **3**, prepared starting from crystals (solid black line) or powders (solid red line).

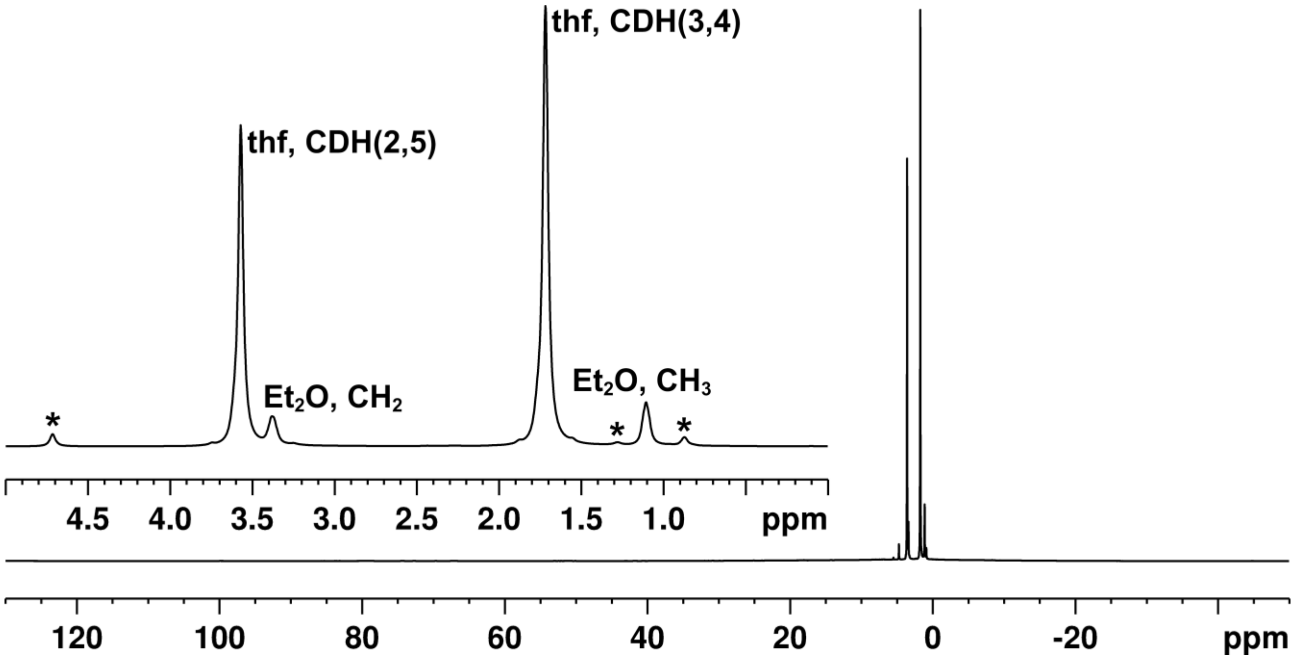

Figure S3. ^{1}H NMR spectrum of **3** in thf-d_8 (298 K, 400.13 MHz). The inset shows a magnification of the high-field region. The asterisks mark impurity signals. The singlet at 4.72 ppm is an unknown trace impurity in thf-d_8, while those at 1.28 (br, CH_2) and 0.88 (br, CH_3) ppm arise from traces of grease.

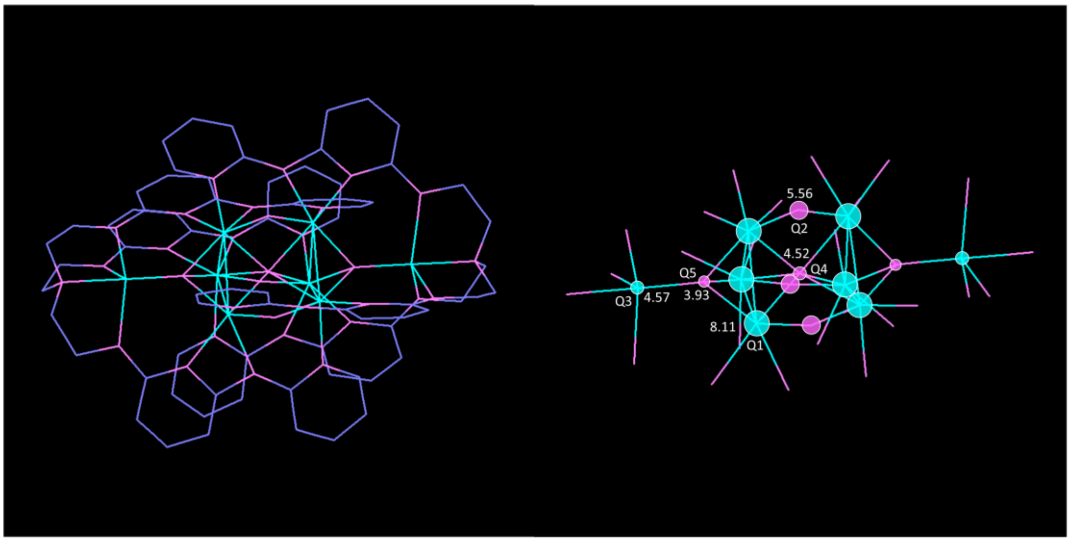

Figure S4. Molecular structure of **3** (wireframe model), as obtained in the early stages of refinement. H atoms are omitted for clarity, along with C atoms in the structure on the right. The circles on the right represent the residual electron density (Q-peaks, in eÅ^{-3}) resulting from a Fourier synthesis with phases based on the organic ligands alone.

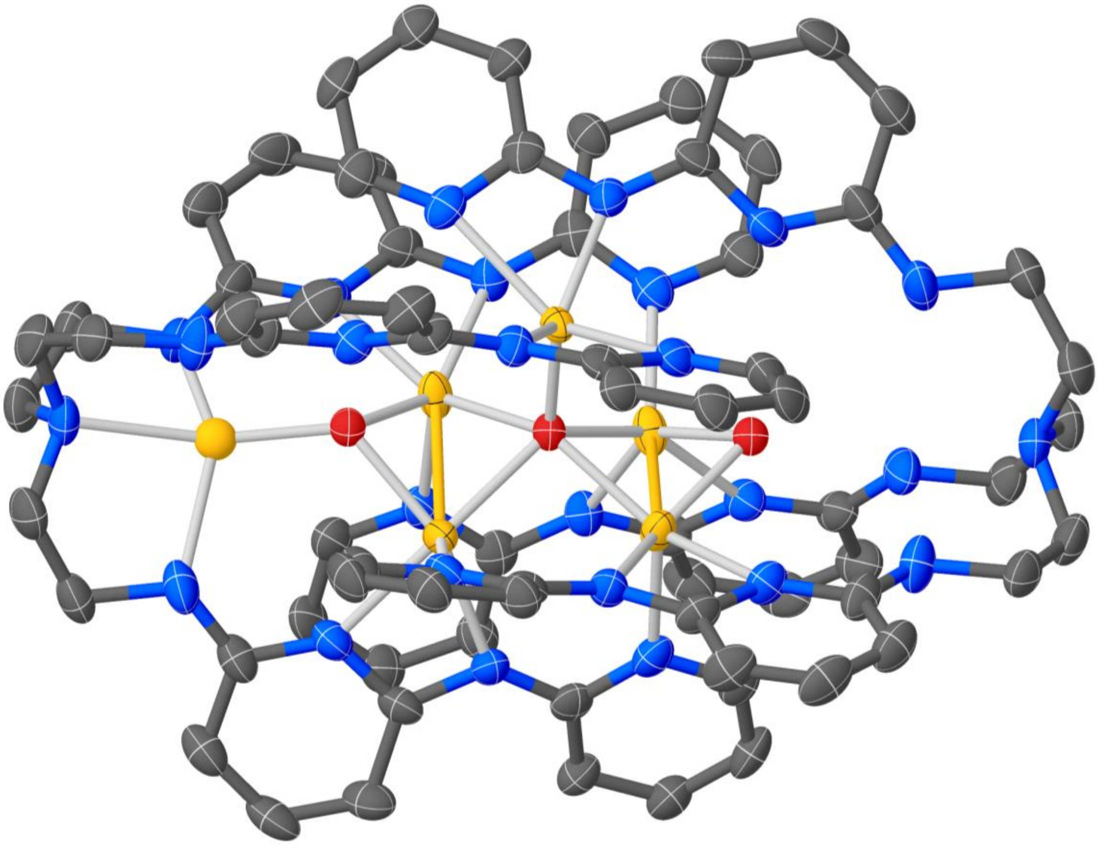

Figure S5. Molecular structure of **3**. H atoms are omitted for clarity and displacement ellipsoids are plotted at 40% probability level. Color code: orange, Fe; red, O; blue, N; dark gray, C.

Table S1. Crystal data and refinement parameters for **3**.

Compound	$[Fe_6O_2(OH)(H_3L)L]$
Chemical formula	$C_{72}H_{70}Fe_6N_{26}O_3$
Formula weight	1682.64
T (K)	115(2)
Crystal size (mm^3)	0.20 × 0.09 × 0.03
Radiation	Mo-Kα (λ = 0.71073 Å)
Crystal system	trigonal
Space group	$P\bar{3}1c$
a (Å)	13.9467(7)
b (Å)	13.9467(7)
c (Å)	23.1917(13)
α (deg)	90
β (deg)	90
γ (deg)	120
V (Å^3)	3906.7(4)
Z	2
ρ_{calcd} (g cm^{-3})	1.430
$2\theta_{min}/2\theta_{max}$ (deg)	6.818/50.004
Reflections collected/independent	18337/2301
R_{int}	0.0763
No. of parameters/restraints	171/0
$R1/wR2$ (all data)	0.1351/0.2983
$R1/wR2$ ($I \geq 2\sigma(I)$)	0.0851/0.2453
GOF	1.049
Largest diff. peak/hole (eÅ^{-3})	2.04/−0.81

Table S2. Results of BVS calculations on **3**.[a]

Atom	BVS(Fe^{2+})	BVS(Fe^{3+})	BVS(Fe^{2+})[b]
Fe1	1.898	2.143	1.994
Fe2	2.008	2.299	2.136
Fe3	1.840	2.087	1.910
O1	1.949	2.085	
O2 with/without Fe3	1.711/0.982	1.831/1.051	

[a]Unless otherwise noted, bond-valence parameters (R_0; B) were taken from file bvparm2020.cif available at https://www.iucr.org/resources/data/datasets/bond-valence-parameters: Fe^{2+}−N^{3-} (1.76 Å; 0.37 Å), Fe^{3+}−N^{3-} (1.82 Å; 0.37 Å), Fe^{2+}−O^{2-} (1.734 Å; 0.37 Å), Fe^{3+}−O^{2-} (1.759 Å; 0.37 Å). Only bond distances within 3.0 Å were included in the calculation. [b]Calculated using B = 0.37 Å and R_0 = 1.792 Å for Fe^{2+}−N^{3-} bonds with all nitrogen donors except N1. This specialized value of R_0 provides the best least-squares (LS) fit of calculated BVS to nominal atomic valence in 35 HS Fe^{2+} complexes with N_{py} and N_{am} donors from dpa^{-} or $tpda^{2-}$ ligands (see Figure S6).

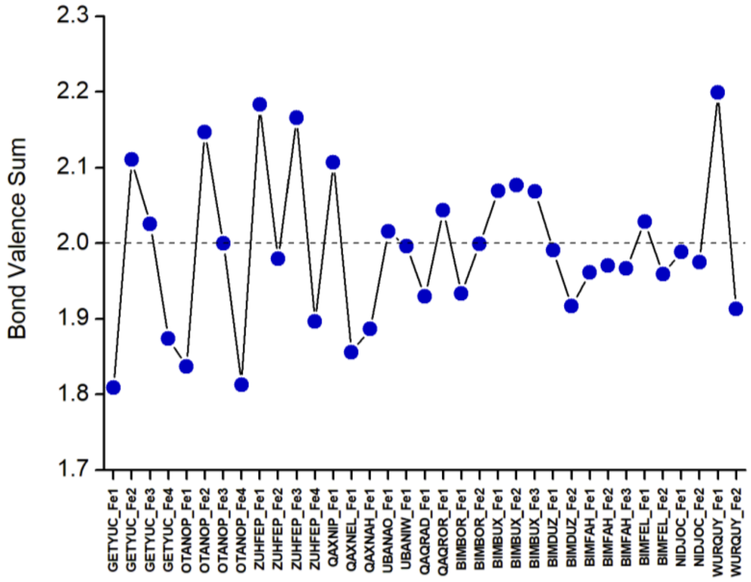

Figure S6. BVS values for 35 HS Fe^{2+} complexes with dpa^{-} or $tpda^{2-}$ ligands appearing in 17 CCDC entries (GETYUC,[1] OTANOP,[2] ZUHFEP,[3] QAXNIP,[4] QAXNEL,[4] QAXNAH,[4] UBANAO,[5] UBANIW,[5] QAQRAD,[6] QAQROR,[6] BIMBOR,[7] BIMBUX,[7] BIMDUZ,[7] BIMFAH,[7] BIMFEL,[7] NIDJOC,[7] and WURQUY[8]), calculated using B = 0.37 Å and R_0 = 1.792(5) Å for Fe^{2+}–N^{3-} bonds with dpa^{-} or $tpda^{2-}$ nitrogen donors (other structures having disordered metal atom positions were disregarded). This R_0 value provides the best LS fit to the nominal atomic valence (dashed line). Any other bond type was modelled using B = 0.37 Å and a standard R_0 value from bvparm2020.cif, namely 2.06 Å for Fe^{2+}–Cl^{-}, 2.21 Å for Fe^{2+}–Br^{-}, 1.734 Å for Fe^{2+}–O^{2-}, and 1.76 Å for Fe^{2+}–N^{3-} bonds with bis(trimethylsilyl)amido ligands. Only bond distances within 3.0 Å were included in the calculation. In the last 11 CCDC entries, the contribution of Fe^{2+}–M interaction (M = Cr^{2+}, Mo^{2+}, W^{2+}, Ni^{2+}, Pd^{2+}, Pt^{2+}, Fe^{2+}) was treated as an additional LS parameter and estimated as 0.16(3) valence units (vu). The root-mean-square-deviation of the data is 0.10 vu.

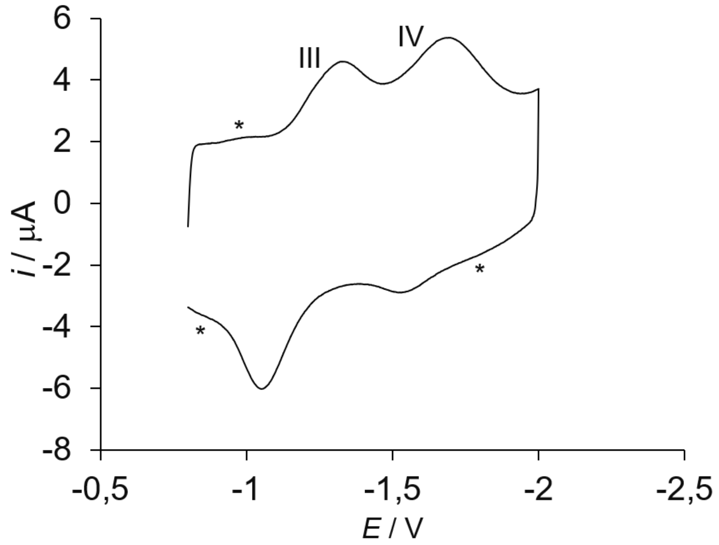

Figure S7. CV of **3** (0.25 mM in thf) at –10 °C. GC working electrode, base electrolyte 0.05 M TBATFB, scan rate 0.5 V s^{-1}, potential range –0.75/–2 V vs. Fc^{+}/Fc reference. The signals marked with an asterisk have very low peak currents and confidently arise from minor additional species.

Table S3. Molar conductivity of **3** at different temperatures.

T / °C	Λ_M / ohm^{-1} cm^2 mol^{-1} [b]
–23	0.090
–15	0.092
–10	0.094
–5	0.097
2	0.101

[a]Experimental conditions: 0.25 mM in thf. [b]The estimated uncertainty is ±0.008 ohm^{-1} cm^2 mol^{-1}.

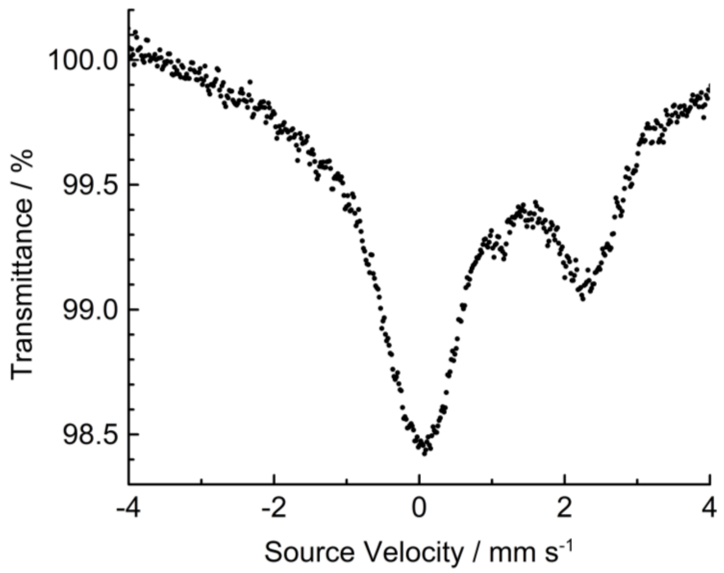

Figure S8. Mössbauer spectrum of **3** at 10 K.

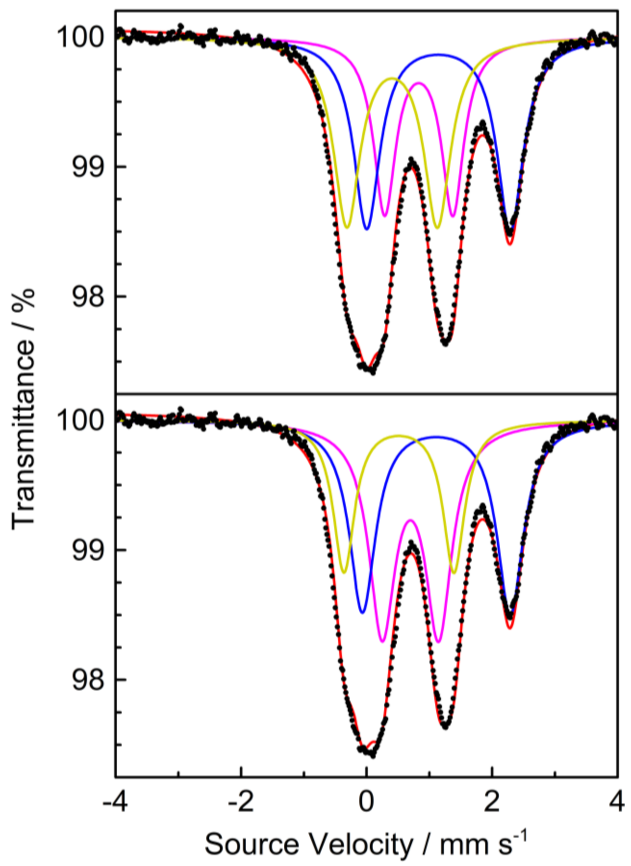

Figure S9. Mössbauer spectrum of **3** at 77 K (solid dots) with accompanying **FitS1** (top) and **FitS2** (bottom) as red lines. The red traces are additionally divided into three quadrupole doublets (purple, yellow, and blue lines).

Table S4. Parameters obtained in the fit of the Mössbauer spectrum of **3** at 77 K.

	Contribution	δ (mm s^{-1})	ΔE_Q (mm s^{-1})	Linewidth (mm s^{-1})	Relative Area
**Fit1**	1	0.70	1.12	0.63	0.69
	2	0.88	2.60	0.32	0.13
	3	1.10	2.60	0.37	0.18
**Fit2**	1	0.70	1.11	0.63	0.69
	2	1.00	2.78	0.38	0.19
	3	1.00	2.34	0.30	0.12
**Fit3**	1	0.71	1.09	0.56	0.67
	2	0.89	2.56	0.32	0.17
	3	1.11	2.57	0.31	0.17
**Fit4**	1	0.71	1.09	0.56	0.67
	2	1.00	2.34	0.32	0.17
	3	1.01	2.79	0.31	0.17
**FitS1**	1	0.83	1.09	0.43	0.28
	2	1.15	2.29	0.51	0.36
	3	0.41	1.44	0.51	0.36
**FitS2**	1	0.70	0.90	0.51	0.40
	2	1.11	2.36	0.51	0.36
	3	0.51	1.75	0.41	0.24

Note on the fitting of magnetic susceptibility data. In order to estimate the magnitude of magnetic couplings in **3**, the results of BVS analysis and DFT calculations (*e.g.* spin population data, predicted Mössbauer parameters, and calculated J-values) were used to fit the experimental $\chi_M T$ vs. T curve (Figure 5) based on a simple Heisenberg Hamiltonian for localized spins (Eq. S1; see Figure 6 for the site numbering scheme). Magnetic anisotropy was neglected in this highly simplified model.

$$\hat{H} = -2J_a(\hat{\mathbf{S}}_1 \cdot \hat{\mathbf{S}}_3 + \hat{\mathbf{S}}_1 \cdot \hat{\mathbf{S}}_4 + \hat{\mathbf{S}}_2 \cdot \hat{\mathbf{S}}_3 + \hat{\mathbf{S}}_2 \cdot \hat{\mathbf{S}}_4 + \hat{\mathbf{S}}_3 \cdot \hat{\mathbf{S}}_4) - 2J_b(\hat{\mathbf{S}}_2 \cdot \hat{\mathbf{S}}_{56} + \hat{\mathbf{S}}_3 \cdot \hat{\mathbf{S}}_{56} + \hat{\mathbf{S}}_4 \cdot \hat{\mathbf{S}}_{56}) + g\mu_B \mathbf{B} \cdot \hat{\mathbf{S}} \tag{S1}$$

In Eq. S1, $\mathbf{S}_i$ is the spin vector at the i-th site, $\mathbf{S}_{56}$ is $\mathbf{S}_5 + \mathbf{S}_6$, J_a and J_b are the exchange coupling constants appearing in Figure S10, μ_B is the Bohr magneton, **S** is the total spin vector, **B** is the applied magnetic field, and a unique g factor was assigned to all metal sites. Notice that positive and negative J-values indicate ferro- and antiferromagnetic interactions, respectively. The rationale behind Eq. S1 is as follows. If metal sites 5 and 6 are taken as HS Fe^{2+} and their spins are ferromagnetically coupled with $J_{56} = 67$ cm^{-1}, as suggested by DFT calculations, the ground $S_{56} = 4$ state of the pair is separated from the first-excited $S_{56} = 3$ state by more than 500 cm^{-1}. By consequence, the $S_{56} = 4$ state hosts the vast majority of the population even at 300 K (94%) and is thus highly persistent to room temperature. It is then legitimate to assume that spins $\mathbf{S}_5$ and $\mathbf{S}_6$ are rigidly coupled to yield $S_{56} = 4$. Since metal sites 1 and 3 possess the highest iron(III) character, they were assigned $S_1 = S_3 = 5/2$, while sites 2 and 4 were associated with $S_2 = S_4 = 2$. For simplicity, all nearest-neighbor interactions among sites 1, 2, 3, and 4 were described using the same J-value (J_a). Similarly, the interactions between the ferromagnetic pair ($S_{56} = 4$) and each of the sites 2, 3, and 4 were described by a single J-value (J_b). This model, albeit very crude, accounts well for the experimental susceptibility curve of **3** between 5 and 300 K (Figure S10) with the following best-fit parameters (from PHI v3.1.6 software[1]): g = 2.0905(15), J_a = 0.894(11) cm^{-1}, and J_b = −0.355(3) cm^{-1}. The resulting g factor is consistent with the average g = 2.10 expected for four HS Fe^{2+} ($g \sim 2.15$) and two HS Fe^{3+} ions ($g \sim 2.00$).

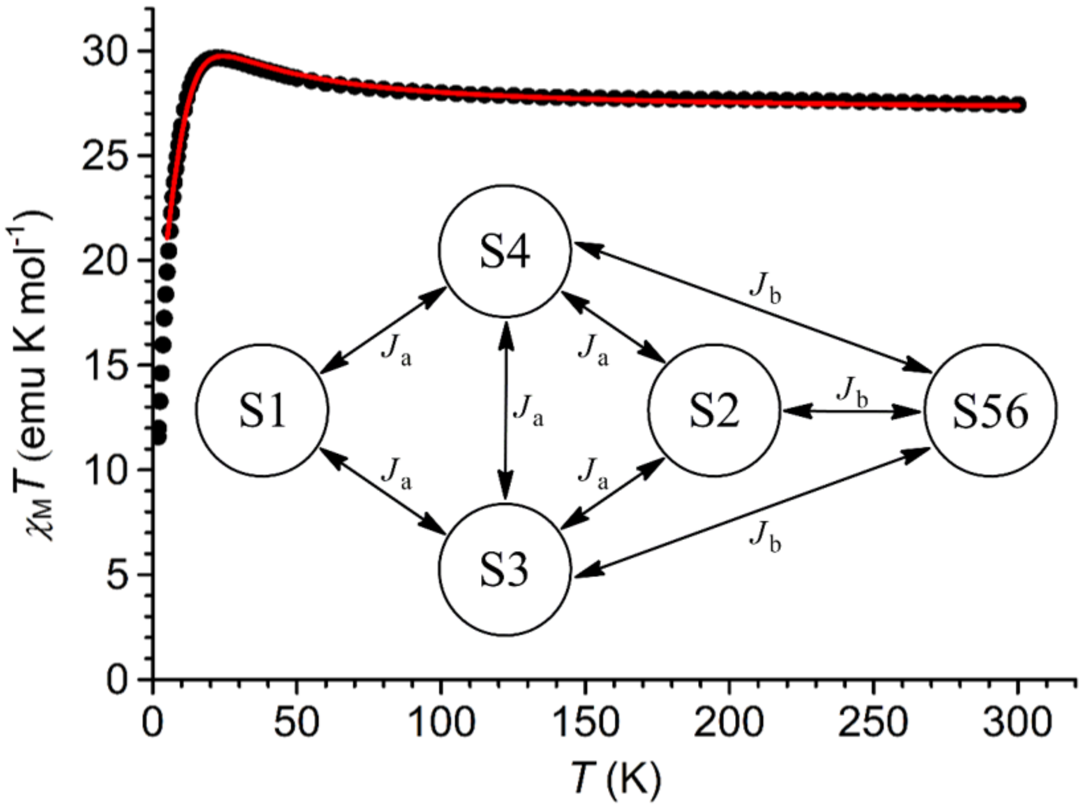

Figure S10. $\chi_M T$ vs. T data for **3** recorded at 1 kOe and schematic representation of the spin model used. The red line provides the best-fit to the experimental data between 5 and 300 K.

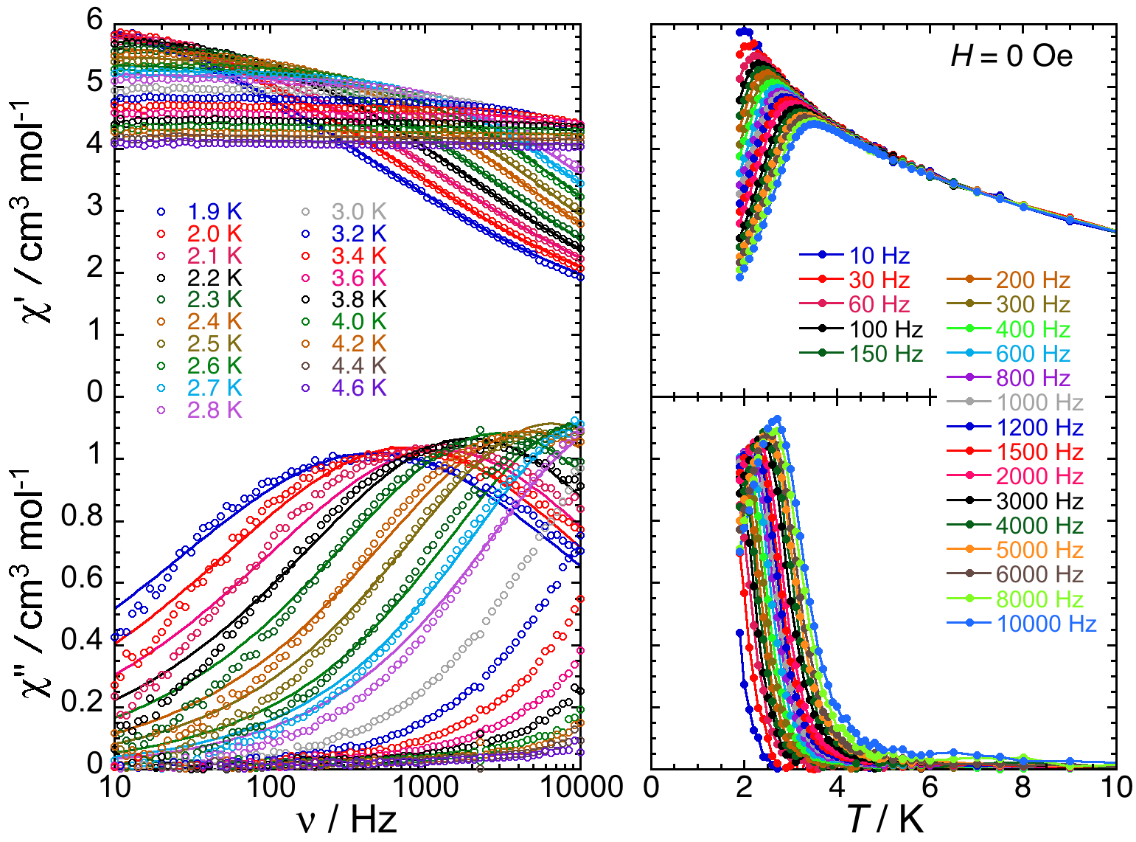

Figure S11. ac frequency (left) and temperature (right) dependences of the real (χ', top) and imaginary (χ'', bottom) parts of the molar ac susceptibility for **3**, for ac frequencies between 10 and 10000 Hz and between 1.9 and 2.8 K. Solid lines on the χ' vs. ν and χ'' vs. ν plots are the generalised Debye fits of the ac data used to extract the temperature dependence of the relaxation time (Figure S15) as well as α, χ_∞' and $\chi_0'-\chi_\infty'$ shown in Figure S12.

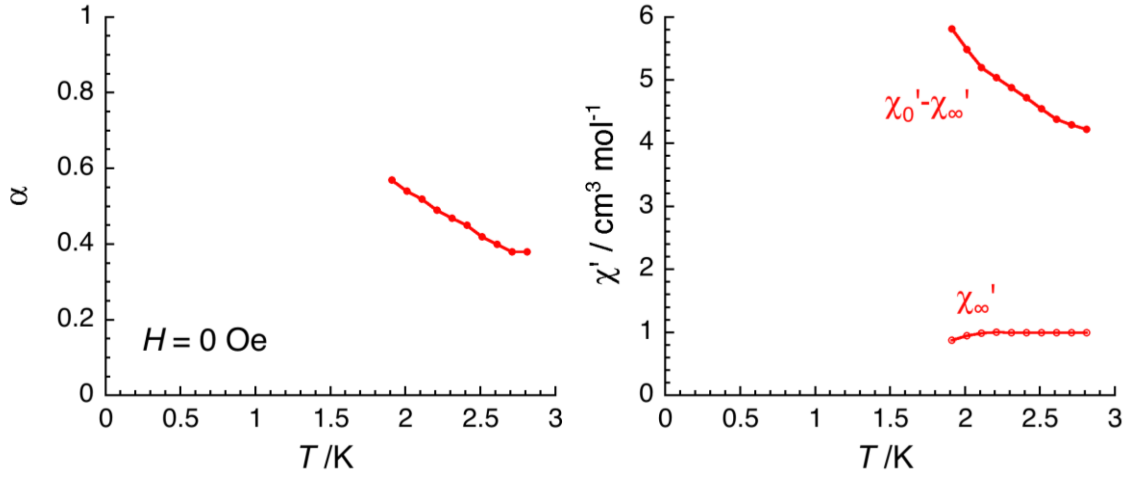

Figure S12. Temperature dependence of the parameters, α, χ_∞' and $\chi_0'-\chi_\infty'$, between 1.9 and 2.8 K at 0 Oe deduced from the generalised Debye fit of the frequency dependence of both real (χ') and imaginary (χ'') components of the molar ac susceptibility shown in Figure S11 for **3**.

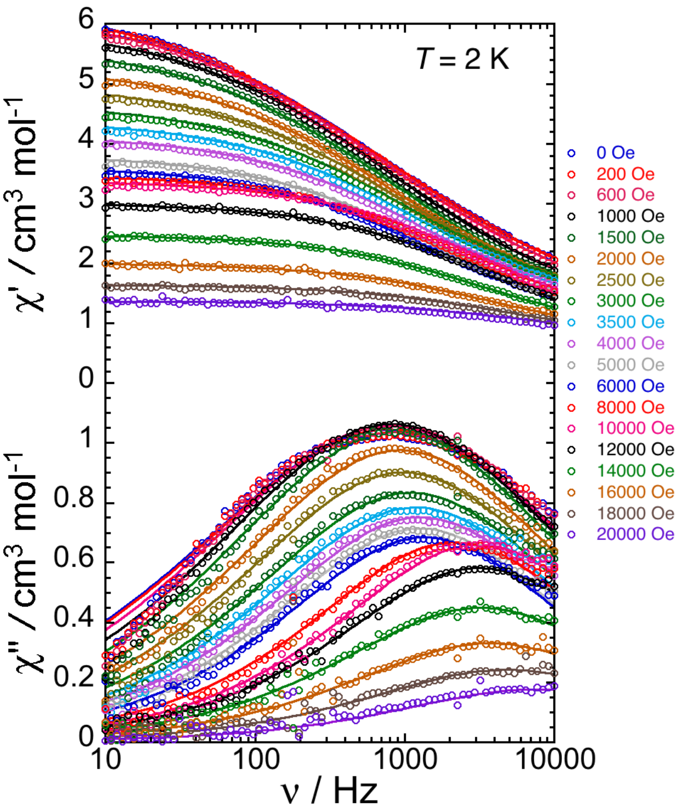

Figure S13. ac frequency dependence of the real (χ', top) and imaginary (χ'', bottom) parts of the molar ac susceptibility for **3**, at 2 K between 10 and 10000 Hz in dc-field between 0 and 20 kOe. Solid lines are the generalised Debye fit of the ac data used to extract the field dependence of the relaxation time shown in Figure S15.

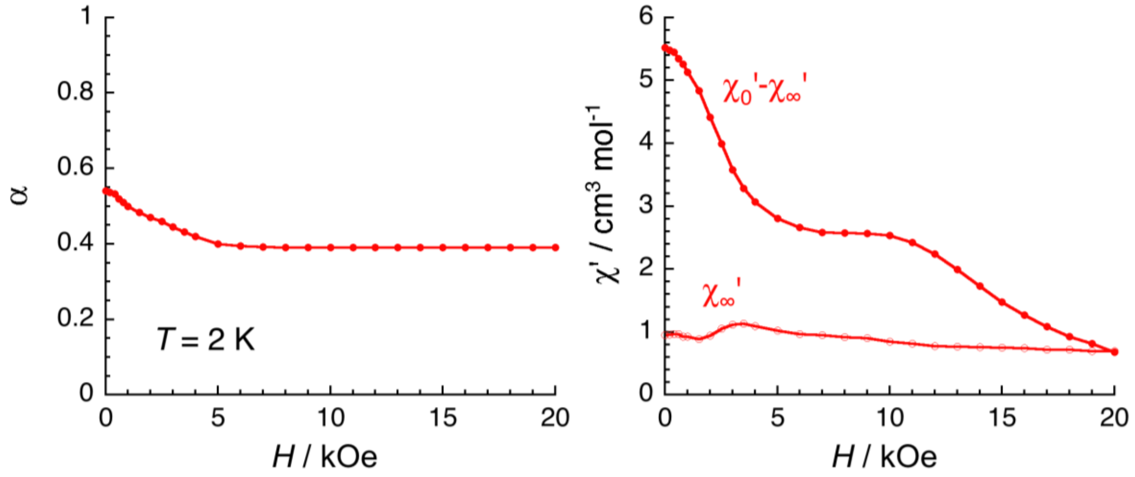

Figure S14. Field dependence of the parameters, α, χ_∞' and $\chi_0'-\chi_\infty'$, between 0 and 20 kOe at 2 K deduced from the generalised Debye fit of the frequency dependence of both real (χ') and imaginary (χ'') components of the molar ac susceptibility shown in Figure S13 for **3**.

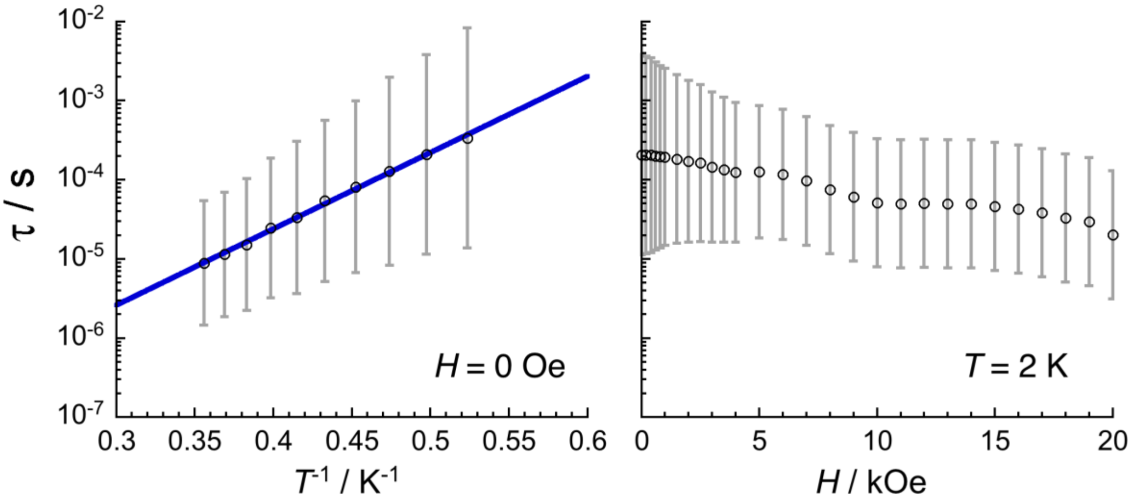

Figure S15. Relaxation time variation for **3** as a function of the temperature at 0 Oe between 1.9 and 2.8 K plotted as τ vs. T^{-1} (left) and as a function of the applied magnetic field at 2 K between 0 and 20 kOe (right) (semi-logarithm plots). The reported relaxation time was estimated from the generalized Debye fits of the molar ac susceptibility data (Figures S11 and S13). Estimated standard deviations of the relaxation time (vertical solid bars) have been calculated from the α parameters of the generalized Debye fit (Figures S12 and S14) and the log-normal distribution as described by Reta and Chilton.[9] The blue line is the best Arrhenius fit discussed in the text.

Table S5. Selected interatomic distances (Å) in **DFT3**.[a]

Fe3–N1	2.30	Fe2–N5	2.25	Fe1–O1	2.07	$Fe1^{III}$–$N5^{I}$	2.09
Fe3–N2	2.04	Fe2–O1	2.01	Fe1–$Fe1^{II}$	2.84	$Fe1^{III}$–$N3^{IV}$	2.10
Fe3–$N2^{I}$	2.15	Fe2–Fe1	3.24	Fe1–$Fe1^{IV}$	3.07	$Fe1^{III}$–O1	2.04
Fe3–$N2^{II}$	2.09	Fe2–$Fe1^{II}$	3.32	$Fe1^{II}$–$N3^{I}$	2.10	$Fe1^{III}$–$O2^{III}$	2.13
Fe3–O2	1.86	Fe2–$Fe1^{III}$	3.44	$Fe1^{II}$–$N5^{IV}$	2.07	$Fe1^{III}$–$Fe1^{IV}$	2.71
Fe3–Fe1	3.44	Fe2–$Fe1^{IV}$	2.81	$Fe1^{II}$–$N4^{I}$	2.10	$Fe1^{IV}$–$N4^{V}$	2.25
Fe3–$Fe1^{II}$	3.51	Fe1–$N3^{II}$	2.14	$Fe1^{II}$–O2	1.95	$Fe1^{IV}$–$N5^{II}$	2.19
Fe2–$N5^{III}$	2.17	Fe1–$N5^{V}$	2.04	$Fe1^{II}$–O1	2.15	$Fe1^{IV}$–$N3^{V}$	2.13
Fe2–N4	2.02	Fe1–$N4^{II}$	2.13	$Fe1^{II}$–$Fe1^{III}$	2.95	$Fe1^{IV}$–O1	2.08
Fe2–$N4^{III}$	2.06	Fe1–O2	1.95	$Fe1^{III}$–$N4^{IV}$	2.19	$Fe1^{IV}$–$O2^{III}$	2.12

[a]For the atom numbering scheme, see Figure 1.

Table S6. Spin population analysis of **DFT3.**

Metal site[a]	Mulliken spin population	Loewdin spin population	Natural spin population
1	3.93	3.89	3.55
2	3.77	3.71	3.50
3	3.81	3.79	3.53
4	3.82	3.78	3.52
5	3.72	3.68	3.42
6	3.64	3.64	3.31

[a]For the labelling of metal sites, see Figure 6.

Table S7. Quadrupole splitting values (mm s^{-1}) in **3** as obtained from experiment (**Fit3** and **Fit4**) and from model **DFT3**.

ΔE_Q from **Fit3** and **Fit4**[a]	Metal site[b]	ΔE_Q from **DFT3**
1.09–1.11 (4)<br>2.34–2.57 (1)<br>2.56–2.79 (1)	1	0.82
	2	0.78
	3	0.86
	4	1.83
	5	0.83
	6	0.70

[a]Numbers in paretheses are the relative areas of the three subspectra. [b]For the labelling of metal sites, see Figure 6.

Table S8. Mayer bond orders for Fe–Fe interactions in **DFT3**.[a]

Metal sites	*MBO* from **DFT3**
2–3	0.14
2–6	0.45
3–4	0.16
3–6	0.20
4–5	0.31
5–6	0.35

[a]Only bond orders ≥ 0.1 are listed.

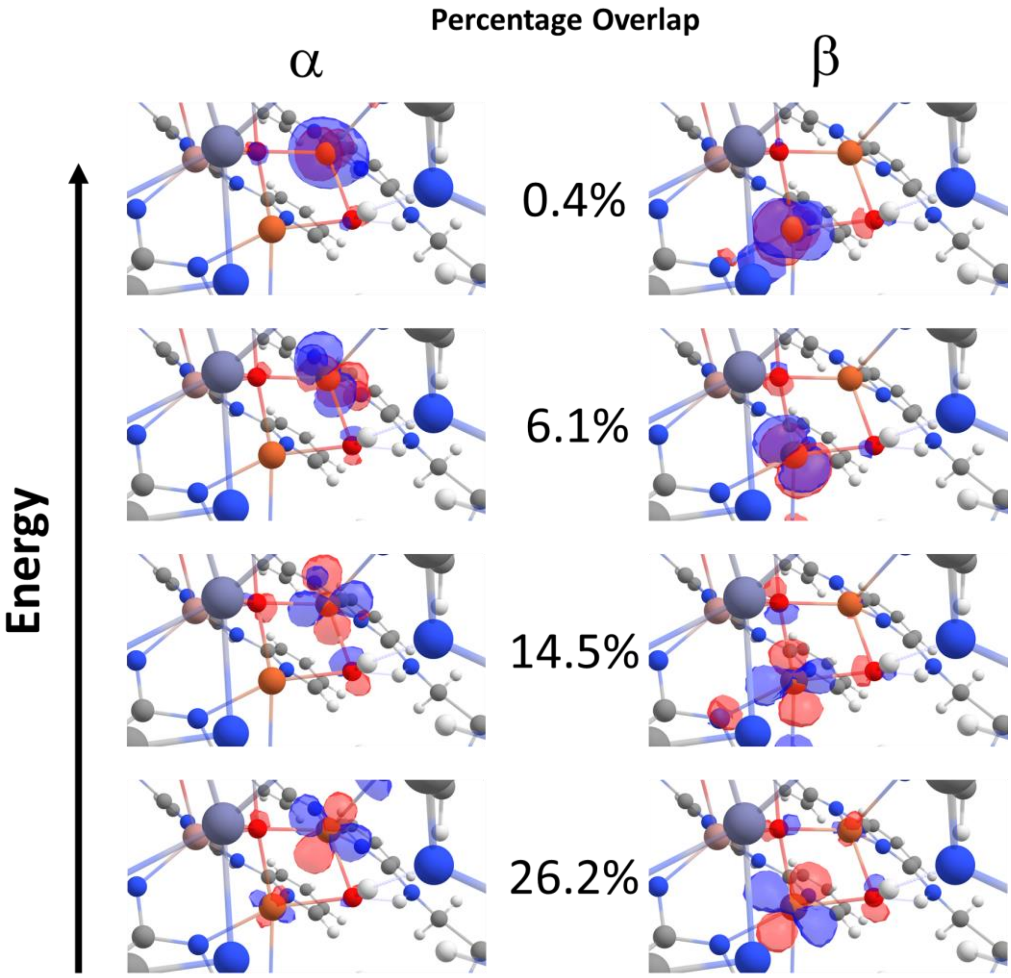

Figure S16. Molecular orbitals involved in the magnetic exchange in **DFT4.** For clarity, the major contributions on the Fe and O atoms were chosen as the view of these MOs to display. Orbitals were visualized using Chemcraft contour value of 0.05. Atomic coloring scheme: orange, Fe; red, O; dark blue, N; white, H; grey, C; light blue, Zn; light red, Ga.

References

(1) Nicolini, A.; Galavotti, R.; Barra, A.-L.; Borsari, M.; Caleffi, M.; Luo, G.; Novitchi, G.; Park, K.; Ranieri, A.; Rigamonti, L.; Roncaglia, F.; Train, C.; Cornia, A. Filling the Gap in Extended Metal Atom Chains: Ferromagnetic Interactions in a Tetrairon(II) String Supported by Oligo-α-Pyridylamido Ligands. *Inorg. Chem.* **2018**, *57* (9), 5438–5448. https://doi.org/10.1021/acs.inorgchem.8b00405.

(2) Nicolini, A.; Affronte, M.; SantaLucia, D. J.; Borsari, M.; Cahier, B.; Caleffi, M.; Ranieri, A.; Berry, J. F.; Cornia, A. Tetrairon(II) Extended Metal Atom Chains as Single-Molecule Magnets. *Dalton Trans.* **2021**, *50* (22), 7571–7589. https://doi.org/10.1039/D1DT01007G.

(3) Sundberg, J.; Vad, M. S.; McGrady, J. E.; Björemark, P. M.; Håkansson, M.; McKenzie, C. J. Accessing Iron Amides from Dimesityliron. *J. Organomet. Chem.* **2015**, *786*, 40–47. https://doi.org/10.1016/j.jorganchem.2015.03.015.

(4) Maddock, L. C. H.; Borilovic, I.; McIntyre, J.; Kennedy, A. R.; Aromí, G.; Hevia, E. Synthetic, Structural and Magnetic Implications of Introducing 2,2′-Dipyridylamide to Sodium-Ferrate Complexes. *Dalton Trans.* **2017**, *46* (20), 6683–6691. https://doi.org/10.1039/C7DT01319A.

(5) Nippe, M.; Bill, E.; Berry, J. F. Group 6 Complexes with Iron and Zinc Heterometals: Understanding the Structural, Spectroscopic, and Electrochemical Properties of a Complete Series of M≣M···M′ Compounds. *Inorg. Chem.* **2011**, *50* (16), 7650–7661. https://doi.org/10.1021/ic2011315.

(6) Nippe, M.; Turov, Y.; Berry, J. F. Remote Effects of Axial Ligand Substitution in Heterometallic Cr≣Cr···M Chains. *Inorg. Chem.* **2011**, *50* (21), 10592–10599. https://doi.org/10.1021/ic2011309.

(7) Liu, Y.; Hua, S.; Cheng, M.; Yu, L.; Demeshko, S.; Dechert, S.; Meyer, F.; Lee, G.; Chiang, M.; Peng, S. Electron Delocalization of Mixed-Valence Diiron Sites Mediated by Group 10 Metal Ions in Heterotrimetallic Fe-M-Fe (M=Ni, Pd, and Pt) Chain Complexes. *Chem. Eur. J.* **2018**, *24* (45), 11649–11666. https://doi.org/10.1002/chem.201801325.

(8) Guillet, G. L.; Arpin, K. Y.; Boltin, A. M.; Gordon, J. B.; Rave, J. A.; Hillesheim, P. C. Synthesis and Characterization of a Linear Triiron(II) Extended Metal Atom Chain Complex with Fe–Fe Bonds. *Inorg. Chem.* **2020**, *59* (16), 11238–11243. https://doi.org/10.1021/acs.inorgchem.0c01625.

(9) Reta, D.; Chilton, N. F. Uncertainty Estimates for Magnetic Relaxation Times and Magnetic Relaxation Parameters. *Phys. Chem. Chem. Phys.* **2019**, *21* (42), 23567–23575. https://doi.org/10.1039/C9CP04301B.
